# A favorable follicular helper CD4^+^ T cell programming characterizes neutralization activity in chronic HIV infection

**DOI:** 10.1172/JCI192575

**Published:** 2026-07-14

**Authors:** Eirini Moysi, Ashish A. Sharma, Sijy O’Dell, Spiros Georgakis, Perla Mariana Del Rio Estrada, Ghneim Khader, Alonso Arana, Fernanda Torres-Ruiz, Mauricio González Navarro, Yara Andrea Luna Villalobos, Santiago Avila Rios, Gustavo Reyes-Teran, Margaret H. Beddall, Sung Hee Ko, Frida Belinky, Michail Orfanakis, Laurence de Leval, Ana B. Enriquez, Clarisa M. Buckner, Susan Moir, Helen Lindsay, Raphael Gottardo, Nicole Doria-Rose, Eli A. Boritz, John R. Mascola, Rafick-Pierre Sekaly, Richard A. Koup, Constantinos Petrovas

**Affiliations:** 1 Tissue Analysis Core, Immunology Laboratory, Vaccine Research Center, National Institute of Allergy and Infectious Diseases (NIAID), NIH, Bethesda, Maryland, USA.; 2 Pathology Advanced Translational Research Unit, Department of Pathology, Emory University School of Medicine, Atlanta, Georgia, USA.; 3 Virology Laboratory, Vaccine Research Center, NIAID, NIH, Bethesda, Maryland, USA.; 4 Department of Laboratory Medicine and Pathology, Institute of Pathology, Lausanne University Hospital and University of Lausanne, Lausanne, Switzerland.; 5 Centro de Investigación en Enfermedades Infecciosas, Instituto Nacional de Enfermedades Respiratorias “Ismael Cosío Villegas,” Mexico City, Mexico.; 6 Centro de Investigación en Enfermedades Infecciosas, Instituto Nacional de Enfermedades Respiratorias “Ismael Cosío Villegas,” Subdireccion de Otorrinolaringologia, Instituto Nacional de Rehabilitación “Luis Guillermo Ibarra,” Mexico City, Mexico.; 7 Institutos Nacionales de Salud y Hospitales de Alta Especialidad, Secretaría de Salud de México, Mexico City, Mexico.; 8 ImmunoTechnology Section, Vaccine Research Center,; 9 Virus Persistence and Dynamics Section, Vaccine Research Center, and; 10 Laboratory of Immunoregulation, NIAID, NIH, Bethesda, Maryland, USA.; 11 Biomedical Data Science Center, Lausanne University Hospital and Lausanne University, Lausanne, Switzerland.; 12 ModeX Therapeutics, Weston, Massachusetts, USA.

**Keywords:** AIDS/HIV, Immunology, AIDS vaccine, Adaptive immunity, Cellular immune response

## Abstract

A subset of people living with HIV (PLWH) can produce broadly neutralizing antibodies (bNAbs) against HIV, but the lymph node (LN) dynamics that promote the generation of these Abs are poorly understood. Here, we explored LN-associated histological, immunological, and virological determinants of bNAb generation in a cohort of antiretroviral therapy–naive PLWH. We found that participants who produce bNAbs, termed “neutralizers” (Ns), have a better-preserved LN-associated B cell follicle architecture than do PLWH who do not. The former was associated with a substantially higher in situ prevalence of B-cell lymphoma 6 (Bcl-6^hi^) follicular helper CD4^+^ T cells (Tfh), expressing a molecular program that favors their differentiation and stemness, and substantially reduced IL-10 follicular suppressor CD4^+^ T cells. Furthermore, our data reveal possible molecular targets mediating Tfh–B cell interactions in Ns. Together, we identify germinal center cellular and molecular signatures that could contribute to the development of bNAbs in PLWH.

## Abstract 2

Lymph node determinants associated with the development of neutralizing antibodies in HIV infection.

## Introduction

Chronic HIV infection changes the microarchitecture and cellular composition of lymph nodes (LNs) (1). Microanatomical transformations include disruptions to follicular reticular cell conduits (2) and follicular DC (FDC) networks (3), progressive loss of follicular B cell architecture (4), and alterations in the phenotype and function of T and B cells that can persist despite antiretroviral therapy (ART) (5–8). Presumably, the loss of stromal cells and altered tissue architecture has an important effect on optimal germinal center (GC) development. Follicular helper T cells (Tfh) cells are a subset of CD4^+^ T cells defined by low expression of CCR7 and high expression of the chemokine receptor CXCR5, the costimulatory receptors programmed cell death 1 (PD-1) and ICOS (9), and the lineage commitment transcription factor B-cell lymphoma 6 (Bcl-6) (10). Among other functions, these cells are critical for the initiation and maintenance of germinal center reactions, which is where somatic hypermutation — a process necessary for the generation of high-affinity Abs — takes place (11). Nonhuman primate (NHP) studies of SIV infection have found that Tfh cells can be detected as early as 14 days after challenge (12). Furthermore, CXCR5^hi^PD-1^hi^ CD4^+^ T cells accumulate in the blood and LNs in the chronic stage of the disease in NHPs (13) as well as in humans (14). Despite this expansion of Tfh cells, people living with HIV (PLWH) have diminished neutralizing antibody (NAb) responses against HIV, a profile associated with HIV-associated impairments in Tfh cell function (5, 15). However, whether such functional impairments correlate with altered B cell follicle architectures and to what extent the latter can affect the development of cross-neutralizing and broadly neutralizing antibodies (bNAbs) against the virus itself in chronic HIV disease remain unclear.

The generation of bNAbs is a critical goal of HIV vaccination strategies, as these can neutralize diverse viral isolates. Recent curative research highlights their potential use as therapeutics for HIV treatment (16). These Abs that cross-react with heterologous viruses have been found to arise in a subset of PLWH (10%–30%) following 2–3 years of infection (17), exhibit unusual structural changes to the antigen binding regions and surrounding framework regions, and have a high degree of somatic hypermutation (18–20). High HIV plasma viremia, low total CD4^+^ T cell counts, high frequencies of Tfh cells early in infection, and low frequencies of Tregs have all been associated with the induction of bNAbs in PLWH (21–24). Extrafollicular (Treg) and follicular (Tfr) regulatory CD4^+^ T cells accumulate in the LNs of untreated PLWH and suppress Tfh and B cells, which impair GC reactivity and IgG production (25). How these dynamics translate at the tissue level in the context of chronic HIV viremia in individuals with and without bNAb production, however, remains poorly understood.

In this study, we sought to address these questions by characterizing Tfh cells, Tregs, and overall GC structure in PLWH. We performed a detailed characterization of LN architecture and used flow cytometry, multiparametric confocal imaging, and single-cell RNA-seq (scRNA-seq) to elucidate the virological and immunologic signatures associated with the production of cross-NAbs. Our results revealed that PLWH with high Ab neutralizing activity, termed “neutralizers” (Ns), have higher frequencies of T central memory cells and Tfh cells and unique B cell follicle architecture compared with “non-neutralizers” (NNs). We observed that active, polarized GCs were more frequent in the tissues of Ns and contained transcriptionally distinct Tfh cells that could account for the neutralization activity between the 2 groups of PLWH. Together, our results elucidate potential LN factors that contribute to the generation of NAbs in PLWH. These insights can be used to develop therapeutics and vaccination strategies that promote the generation of bNAbs in PLWH.

## Results

### Determination of plasma neutralizing activity in study participants.

**Figure 1:**
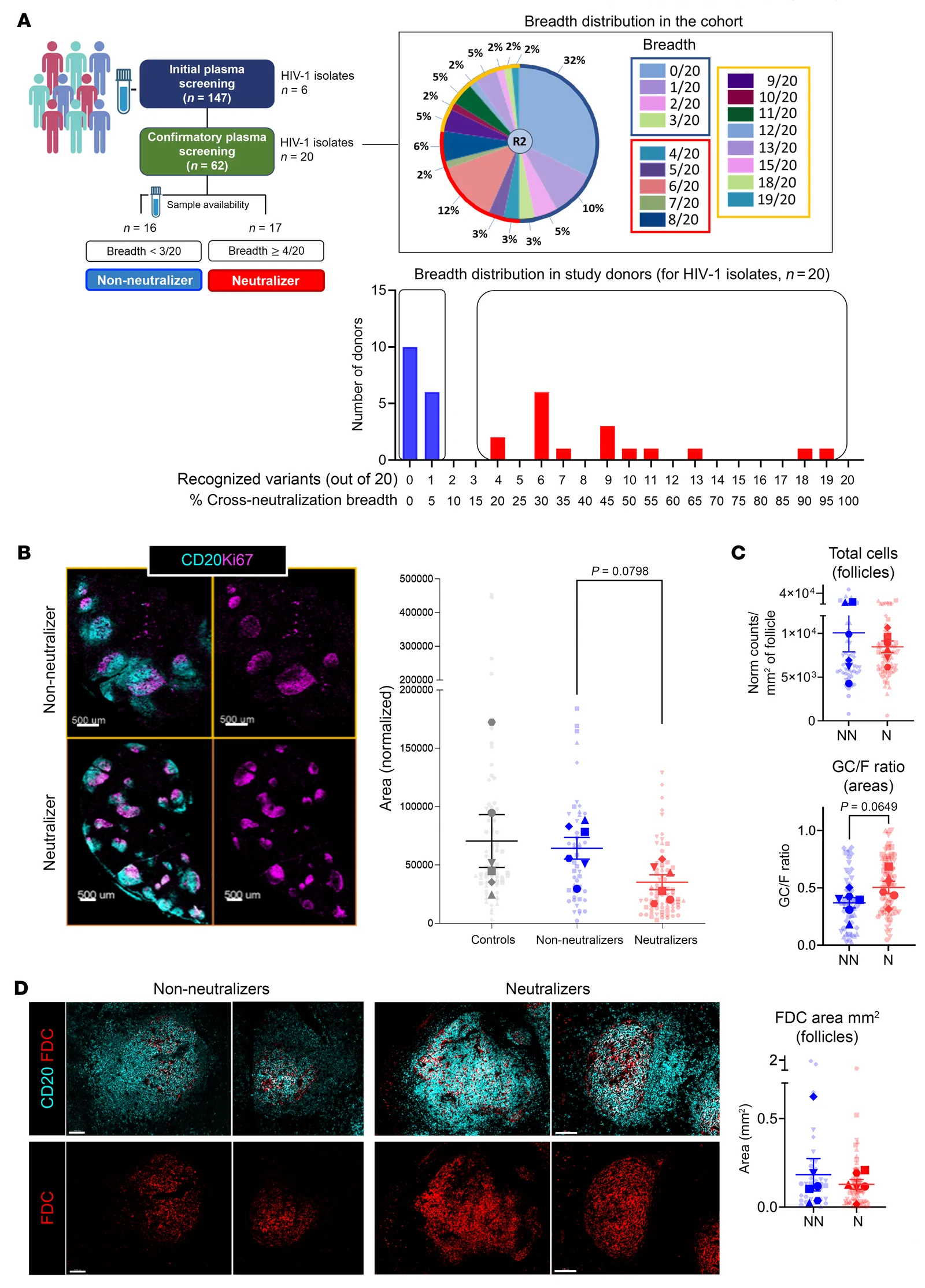
Cross-neutralization is associated with GCs displaying preserved features of maturation. (**A**) Diagram depicting the strategy employed for neutralization screening (left) as well as the distribution of cross-neutralization breadth in the population of study, defined as the percentage of the number of isolates recognized with an ID_50_ of greater than 40 out of 20 isolates tested, in the cohort (pie chart) and among study participants (bar graph). (**B**) Representative confocal images showing the distribution of CD20 (cyan) and Ki67 (magenta) in NNs and Ns. Total B cell follicle area quantification (CD20^hi/dim^) is shown as a superplot of both individual (faint background symbols) and mean ± SEM (bold foreground symbols) follicular measurements in control tissues (*n* = 6, gray), NN tissues (*n* = 6, blue), and N tissues (*n* = 6, red), as measured by quantitative imaging analysis and histocytometry. The different symbols represent different donors. For statistical comparisons using the mean measurements, a Kruskal-Wallis 1-way ANOVA was applied, followed by Dunn’s post hoc test to correct for multiple comparisons. *P* = 0.0798. Original magnification, ×40 (NA 1.3). Scale bars: 500 μm. (**C**) Superplots showing the number of total cells and GC/F ratios (areas) of both individual (faint background symbols) and mean ± SEM (bold foreground symbols) follicular measurements in NNs (*n* = 6, blue) and Ns (*n* = 6, red), as measured by quantitative imaging analysis and histocytometry. Data for each follicle represent total counts of JOJO-1^+^ (a fluorescent nucleic acid stain) events (total cells) normalized (Norm) to the total area (mm^2^) of follicles screened. The different symbols represent different donors. For statistical comparisons using the mean measurements, a Mann-Whitney *U* test was applied. *P* = 0.93; *P* = 0,0649. (**D**) Representative confocal images showing the distribution of FDC (red) in B cell follicles (CD20^dim/hi^ areas, cyan) in NNs (*n* = 6) versus Ns (*n* = 6). Original magnification, ×40. Scale bars: 100 μm), and a Superplot summarizing the FDC areas (mm^2^) measurements of both individual (faint background symbols) and mean ± SEM (bold foreground symbols) follicular measurements in NNs (*n* = 6, blue) and Ns (*n* = 6, red), as measured by quantitative imaging analysis and histocytometry. Different symbols represent different donors. For statistical comparisons using the mean measurements, a Mann-Whitney *U* test was applied. *P* = 0.81.

To determine whether differences exist between LNs, particularly follicles and germinal centers, with neutralizing and non-NAbs, we performed an analysis of cross-neutralizing activity in the serum of a cohort of 147 ART-naive people living with chronic HIV infection. Given the false-positive outcome of the applied neutralization assay for ART samples (26), we avoided using samples from treated PLWH. Participants were identified at the time of diagnosis, and tissues were promptly collected before starting ART. Sera were initially screened against a mini-panel (Supplemental Figure 1A; supplemental material available online with this article; https://doi.org/10.1172/JCI192575DS1) that consisted of the following 6 isolates: Q259.17 (A); Q461.e2 (AD); TH976.17 (AE); YU2.DG (B); CH070.1 (BC); and 0013095-2.11 (C), and findings were then validated for a subset of sera (*n* = 62) with an extended panel of 20 HIV isolates from clades A, AD, AE, AG, B, BC, C, and D. We observed a range of cross-neutralizing activities in the mini-panel screenings. Cross-neutralization of 50% of isolates (3 or more) in 23% of sera was observed, whereas 76% of sera had either low (16%–33% of isolates) or no measurable activity, and we observed a similar pattern in the extended panel screenings. Cross-neutralization of 45% of isolates or more at an ID_50_ of greater than 40 was observed in 25% of sera (high activity), while 26% of sera could cross-neutralize 20%–40% of isolates (medium activity) and 50% of sera could cross-neutralize less than 15% of isolates (low or no activity) (Figure 1A).

On the basis of these findings and on overall tissue and LN cell suspension sample availability, a total of 33 participants with (*n* = 17) or without (*n* = 16) cross-neutralizing activity were selected for further study (Table 1 and Supplemental Tables 1 and 2). Participants were categorized as Ns if their serum neutralizing activity in the extended panel was medium or high (>20% of isolates), or as NNs if their serum neutralization activity was less than 5% (Figure 1A and Supplemental Figure 1B). Since neutralization breadth has been previously shown to depend on the nature of bNAb lineages involved (27), we sought to determine whether known bNAb lineages were represented in the Ns. We performed computational fingerprinting analysis using a neutralization fingerprinting algorithm (NFP) that predicts the epitopes targeted by neutralizing serum by matching patterns of neutralization to those of known bNAbs (28). In the sera from Ns, we detected signal (cutoff 0.25) (29) for 1 or more of the following: VRC01-, 8ANC195-, PGT18- 10E8-, and PGT151-like Abs (Supplemental Figure 1C). Therefore, known bNAb lineages were represented in the serum neutralizing activity in Ns.

### Cross-neutralization is associated with a greater degree of B cell follicle preservation and GC formation.

HIV acquisition causes morphological changes in the microarchitecture of LNs that affect the ability of B cell follicles to perform their function over time (30). Whether reduced cross-neutralization could be associated with anatomical changes is not well understood. Therefore, we examined the features of B cell follicle anatomy that were differentially represented in participants with and without serum cross-neutralizing activity. First, the areas of follicular regions of interest (ROIs), defined by the expression of CD20 (Figure 1B and Supplemental Figure 1D), were evaluated in our cohort and compared with benign HIV-negative participants’ LNs that were characterized by mature follicles (follicular hyperplasia) (Supplemental Table 1). We found that the calculated areas were substantially (*P* = 0.0798) higher in the control and NN participants compared with the N participants, indicating a higher degree of follicular hyperplasia in NNs, in line with previous data (4) (Figure 1B and Supplemental Figure 1D). Although not significant, we found a trend for higher circularity of follicular ROIs in N participants compared with NN participants (Supplemental Figure 1D).

We then evaluated additional elements associated with HIV disease including the overall B cell follicle cellularity, extent of Ki67^hi/lo^CD20^hi/dim^ areas as a surrogate of GCs, and FDC network preservation (31, 32). FDC networks were identified using the anti-CNA.42 Ab, which is highly specific for a fixative-resistant, evolutionarily conserved, glycosylated antigen that is expressed mainly on FDCs (33). We found a similar degree of overall cellularity — defined as the total number of nucleus-positive cells measured within CD20^hi/dim^ B cell follicles — in Ns as compared with NNs (Figure 1C). Next, we examined the extent of GC preservation among tissues. For quantitation, we focused on Ki67, a marker of proliferation and cell-cycle progression (34), as a lack of Ki67 polarization in HIV GCs has previously been shown to associate with GC disorganization (35). Analysis of the area occupied by CD20^hi/dim^Ki67^hi^ B cells (denoted as GCs) versus the CD20^hi/dim^Ki67^hi/lo^ B cell area (denoted here as total follicle [F]) revealed a substantially (*P* = 0.0649) higher ratio of GC/F in Ns than in NNs (*P* = 0.00115) (Figure 1C and Supplemental Figure 1E). Next, we extended our analysis to FDC networks and observed a trend for higher FDC areas in NNs as compared with Ns (Figure 1D) and a reversed trend for the FDC/F ratios (Supplemental Figure 1, F and G). Together, these findings suggest that neutralization activity in chronic HIV is associated with better-preserved follicular structures.

### Cross-neutralization is associated with distinct phenotypic signatures of Tfh cells.

**Figure 2:**
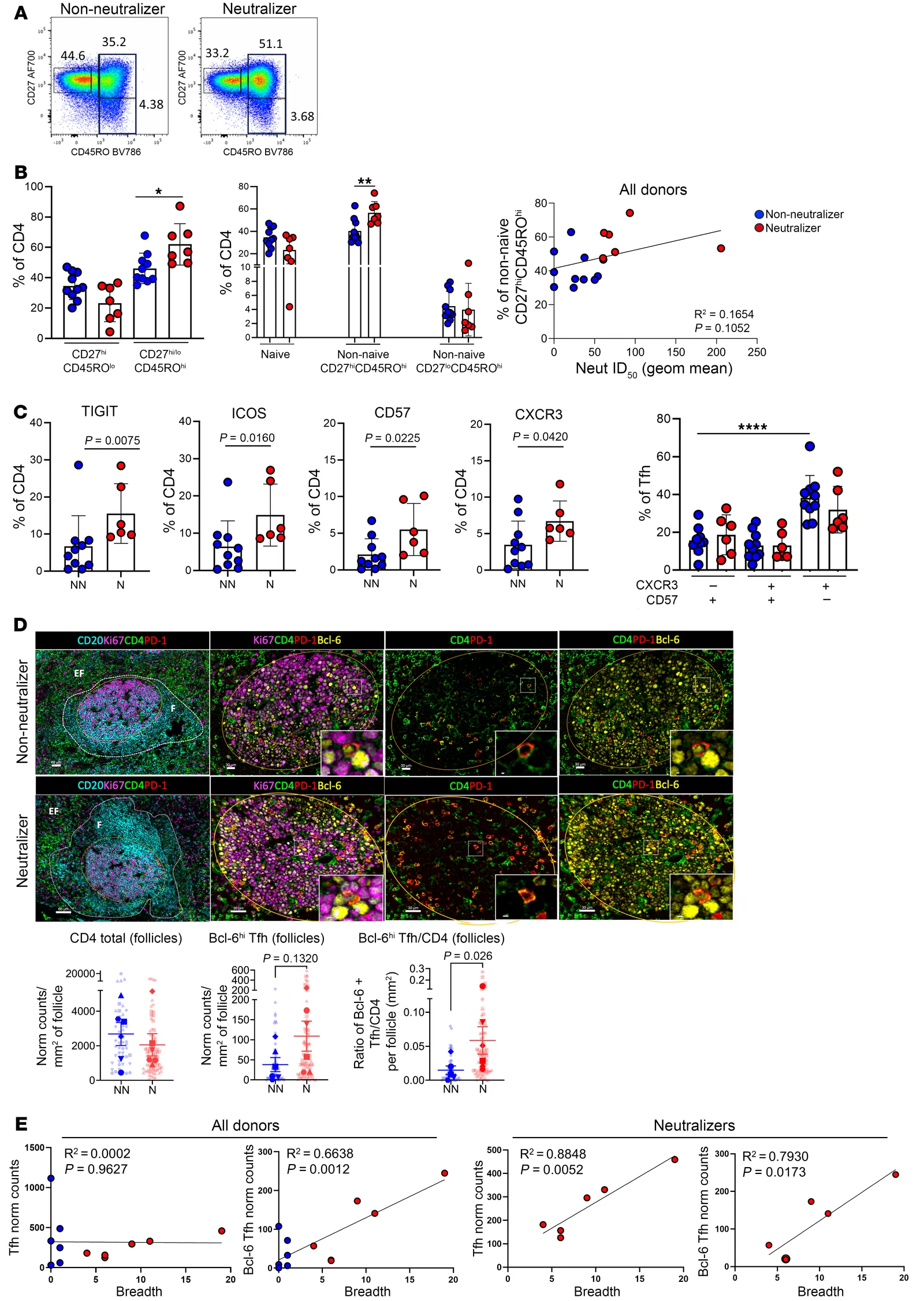
Cross-neutralization is associated with an expansion of the LN CD4^+^ T cell memory compartment and a distinct Tfh cell phenotypic profiling. (**A**) Flow cytometry plots depicting the frequencies of naive and non-naive CD4^+^ T cell subsets in NNs and Ns within the CD3^hi^CD4^hi^ gated populations. (**B**) Graphs showing the relative frequencies of naive and non-naive CD4^+^ T cell subsets in Ns and NNs (left panel) and the positive association (Pearson correlation) between the percentage of non-naive CD27^lo^CD45RO^hi^ CD4^+^ T cells and microneutralization ID_50_ titers as assayed against a 20 HIV isolate panel. (**C**) Cumulative flow cytometry plots showing the frequencies of the receptors TIGIT and ICOS, as well as of the CD57 epitope and CXCR3 within the PD-1^hi^CXCR5^hi^ Tfh cell compartment in NNs and Ns, and frequencies of combined CXCR3 and CD57 expression. TIGIT, *P* = 0.0075; ICOS, *P* = 0.0160; CD57; *P* = 0.0225; CXCR3, *P* = 0.0420 (Mann-Whitney *U* test); NN CXCR5^lo^CD57^hi^ versus NN CXCR3^hi^CD57^lo^, *P* < 0.0001 (1-way ANOVA). ****P < 0.0001. (**D**) Confocal images of follicles (CD20^hi/dim^) and GCs (CD20^hi^Ki67^hi^) in NNs and N showing the distribution of CD4 (green), Tfh (CD4: green, PD-1^hi^: red) and Bcl-6^+^ Tfh (CD4: green, PD-1^hi^: red, Bcl-6^hi^: yellow) within the Ki67 follicular areas (magenta) and Superplots showing total GC-CD4, GC Bcl-6^hi^ Tfh cell densities (cells per mm^2^) and GC Bcl-6^hi^ Tfh/CD4 ratios of both individual (faint background symbols) and mean (bold foreground symbols) follicular measurements (± SEM) in NNs (*n* = 6 blue) versus Ns (*n* = 6 red) as measured by quantitative imaging analysis and histocytometry. Follicular area borders were determined based on the density of CD20 expression, whereas extrafollicular areas (T cell zone) were determined based on the density of CD4. Different symbols represent different donors. For statistical comparisons using the mean measurements, a Mann-Whitney *U* test was applied. *P* = 0.48; *P* = 0.132; *P* = 0.026. Original magnification, ×20. Scale bars: 60 μm left column; 30 μm right columns. (**E**) Regression plots showing the association between bulk Tfh and Bcl-6^hi^ Tfh cells (normalized cell counts adjusted for mm^2^ of area screened) as quantified by confocal imaging analysis and histocytometry, and the breadth of neutralization expressed as the number of HIV isolates recognized among the 20 isolates tested. EF, extrafollicular area; F, follicular area. Mean ± SD values are shown in **B** and **C** and median values in **D**.

Having observed morphological differences in the LNs of NNs and Ns, we next wanted to assess the cellular composition of the LNs in these 2 groups. We performed flow cytometry on single-cell suspensions of LNs and enumerated the frequencies of the following immune subsets: naive (CD27^hi^CD45RO^lo^) and non-naive (CD27^hi^CD45RO^hi^, CD27^lo^CD45RO^hi^) CD4^+^ T cell subsets and PD-1^hi^CXCR5^hi^ Tfh cells (Figure 2A and Supplemental Figure 2A). We found a significantly higher percentage of non-naive CD27^hi/lo^CD45RO^hi^ T cells in N LNs than in NN LNs (*P* = 0.032) (Figure 2B). This was primarily driven by an increase in non-naive CD27^lo^CD45RO^hi^ cells (uO = 0.005) (Figure 2B). Between the latter frequencies and the recorded neutralization potencies, we observed a positive association which, although not significant (*R^2^* = 0.1654, *P* = 0.1052), was suggestive of correlated changes between these 2 factors (Figure 2B).

We found a balanced representation (percentage) of non-naive CD4^+^ T cell subsets defined by the expression of PD-1 and CXCR5 in Ns, while a gradual decrease from non-Tfh cells (PD-1^lo^CXCR5^lo^) to Tfh cells (PD-1^hi^CXCR5^hi^) was observed in NNs (Supplemental Figure 2B). Furthermore, flow cytometric phenotyping of the Tfh cell subset revealed significantly higher expression of several coinhibitory/costimulatory receptors (36–38) in Ns compared with NNs (Figure 2C and Supplemental Figure 2C). With respect to CD57, a marker of highly differentiated GC Tfh cells (39, 40) and CXCR3, a chemokine associated with a Th1-like Tfh cell signature, we found significantly higher frequencies of CD57^lo^CXCR3^hi^ Tfh cells compared with CD57^hi^ CXCR3^lo^ Tfh cells in NNs (*P* < 0.0001), while a trend for higher frequencies of CD57^lo^ CXCR3^hi^ Tfh cells in NNs as compared with Ns was also notable (Figure 2C). Similar findings were also obtained when data from samples with sufficiently representative Tfh cell numbers from NNs and Ns were dissected by *t*-distributed stochastic neighbor embedding (*t*-SNE) analysis (Supplemental Figure 2D). We found a trend for higher representation of CD57^lo^CXCR3^hi^ Tfh cells within the TIGIT^hi^ICOS^hi^CD95^hi^ phenotypic compartment in NNs compared with Ns (cluster/population 5) (Supplemental Figure 2D). On the other hand, a trend for a higher frequency of the cluster bearing the CD57^hi^CXCR3^lo^ signature was seen in Ns (cluster/population 6) (Supplemental Figure 2D). Taken together, our data suggest an altered differentiation profile of Tfh cells in NNs that was characterized by an accumulation of a less differentiated (CD57^lo^), Th1-like (CXCR3^hi^) phenotype in NNs compared with Ns.

We next analyzed the prevalence of Tfh cells within CD20^hi/dim^ follicular areas of the LNs in situ. Using immunofluorescence, we found higher normalized numbers of bulk follicular CD4^+^ T cells in NNs than in Ns (Figure 2D and Supplemental Figure 3A). In contrast to total CD4^+^ T cells, PD-1^hi^Bcl-6^hi^ Tfh cells were substantially (*P* = 0.1320) increased in Ns compared with NNs (Figure 2D and Supplemental Figure 3B). Furthermore, the ratio of Tfh to CD4^+^ T cells within the B cell follicles of Ns was significantly higher than that for NNs (Figure 2D and Supplemental Figure 3B). We also examined whether a correlation existed between the normalized Tfh cell counts and the breadth of serum cross-neutralization. Contrary to PD1^hi^ Tfh cells, we found a significant correlation between PD-1^hi^Bcl-6^hi^ Tfh cells and the cross-neutralization breadth in all participants (*R^2^* = 0.66, *P* = 0.0012) (Figure 2E). We observed a similar profile when the group of Ns was analyzed separately (*R^2^* = 0.79, *P* = 0.0173), which also extended to PD-1^hi^ Tfh cells (*R^2^* = 0.88, *P* = 0.0052) (Figure 2E). Overall, these results show that the development of cross-neutralizing breadth was associated with a higher in situ prevalence of PD-1^hi^ and “effector” Bcl-6^hi^ Tfh cells.

### Cross-neutralization is associated with a molecular profile favoring the development and function of Tfh cells.

**Figure 3:**
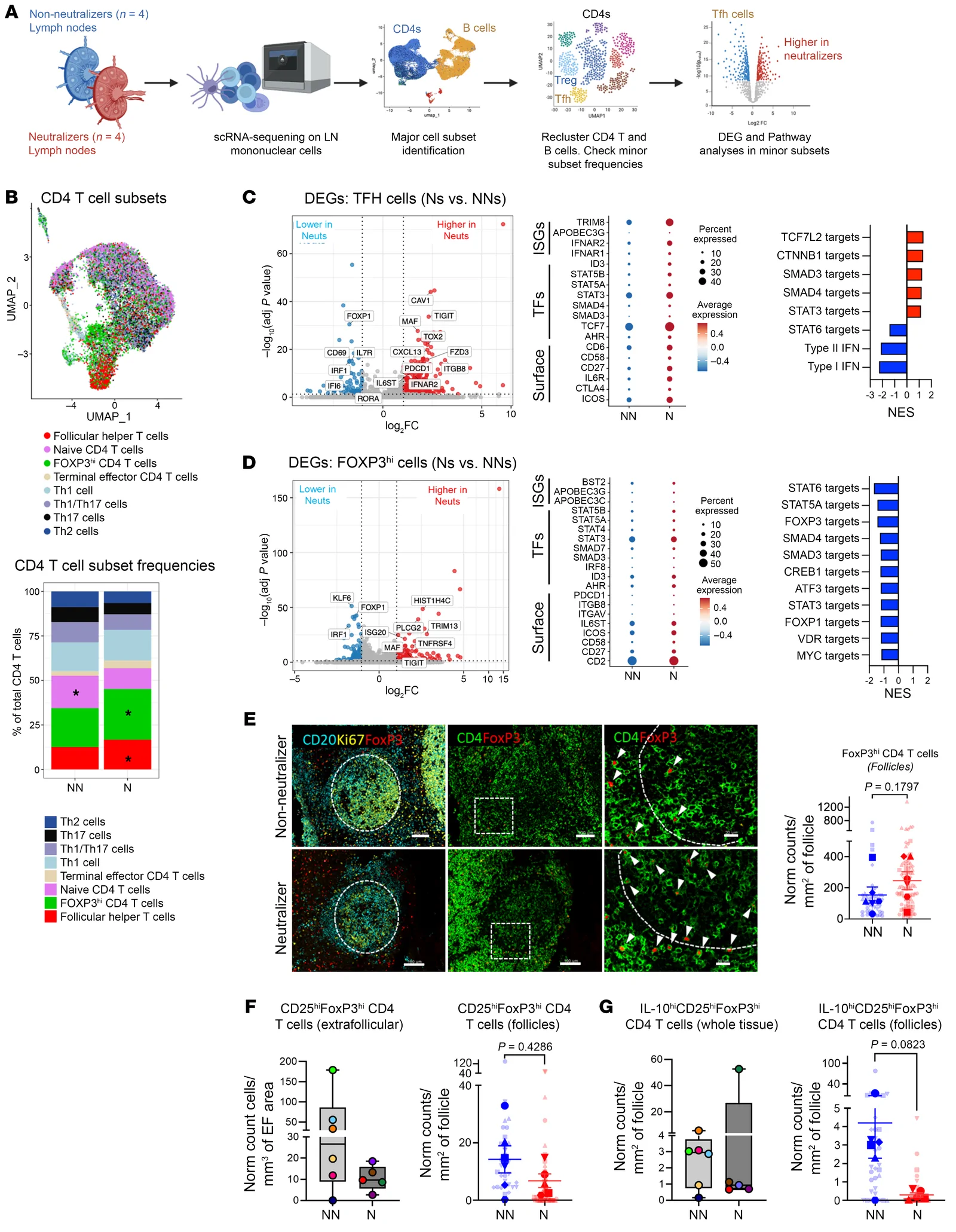
Tfh cells are characterized by a molecular profile favoring their differentiation and longevity in cross-neutralization participants. (**A**) scRNA analysis workflow. DEG, differentially expressed gene. (**B**) UMAP projections of total CD4^+^ T cells with color-coded cell populations and CD4^+^ T cell subpopulations identified and their percentages in NNs versus Ns. (**C** and **D**) Volcano plots and dot plots are shown to illustrate transcriptomic differences and pathway enrichment between Ns (Neut) and NNs (NonNeut). DEGs were identified using the MAST (hurdle model–based) test implemented in the FindMarkers function of the Seurat package in R. *P* values were adjusted by the Benjamini-Hochberg method, and genes meeting an adjusted *P* < 0.05 and |log_2_fold change [FC]| > 0.5 are highlighted in the volcano plots. Blue and red dots in the volcano plots represent genes that are significantly downregulated or upregulated in Ns, respectively. The accompanying dot plots depict average scaled expression (*z* scores) and the percentage of cells expressing each gene within Tfh or Treg subsets. Color intensity reflects relative expression, while dot size indicates the fraction of expressing cells. NESs from the GSEA are shown separately for selected pathways and transcription factor target gene sets. (**E**) Representative confocal images showing FOXP3^hi^ CD4^+^ T cells in extrafollicular and follicular areas in NNs versus Ns. FoxP3^hi^ CD4^+^ T cells (white arrows) were defined by means of concurrent CD4 (green) and FoxP3 (red) expression within CD20 (cyan) and Ki67 (yellow) double-positive areas. Original magnification, ×40; scale bars: 100 μm and 20 μm (enlarged images corresponding to the white dotted enclosures). Superplots showing the normalized per area imaged FoxP3^hi^ CD4^+^ T cell numbers of both individual (faint background symbols) and mean (bold foreground symbols) follicular measurements (± SEM) in NNs (*n* = 6, blue) compared with Ns (*n* = 6, red), as measured by quantitative imaging analysis and histocytometry. The different symbols represent different donors. For statistical comparisons using the mean measurements, a Mann-Whitney *U* test was used. *P* = 0.1797. Superplots of CD25^hi^FoxP3^hi^ (**F**, right) and IL-10^hi^CD25^hi^FoxP3^hi^ (**G**, right) CD4^+^ T cells of both individual (faint background symbols) and mean (bold foreground symbols) follicular measurements in NNs (*n* = 6, blue) versus Ns (*n* = 5, red), as measured by quantitative imaging analysis and histocytometry. The different symbols represent different donors. For statistical comparisons using the mean measurements, a Mann-Whitney *U* test was applied (*P* = 0.426, *P* = 0.0823). Dot plots for extrafollicular (**F**, left) and whole tissue (**G**, left) data, where each participant tissue is represented by a circle of a different color. Mean values are shown in dot plots (**E**–**G**) and quartiles in box plots (**F** and **G**).

To identify molecular effector pathways that could trigger the functional differences observed when comparing Tfh cells from N and NN groups, we performed 10X scRNA-seq on LN-derived cells (Figure 3A). Analysis of lineage biomarkers enabled the identification of the main immune cell types (Supplemental Figure 4A). Further clustering was performed on the basis of the expression of CD4^+^ T cell–specific genes (Supplemental Figure 4, B and C). We observed a significantly higher frequency of Tfh cells in Ns, whereas the frequency of naive CD4^+^ T cells was significantly higher in NNs than in Ns (Figure 3B), which corroborates our flow cytometric data (Figure 2). We then focused on characterizing the Tfh cell compartment. We observed higher transcription of genes encoding critical regulators that promote Tfh cell differentiation (e.g., *MAF*, *IL6ST*, *PDCD1*, *ICOS*, and *TOX2*), stemness (*TCF7*), and interaction with GC B cells (*CXCL13*) in Ns compared with NNs (Figure 3C). Interestingly, Tfh cells from Ns had a significant upregulation of Wnt signaling pathways as well as of targets that are downstream of TCF7L2, SMAD3,4 and STAT3, whereas NN Tfh cells were associated with significant upregulation of type I and II IFN signaling and STAT6 target genes. All shown pathways were significantly different between the groups (*P* < 0.05) (Figure 3C). Of note, Tfh cells from Ns exhibited significantly lower expression of the hallmark apoptosis gene set (normalized enrichment score [NES] = 1.32, *P* = 0.05), driven by leading-edge genes such as *TXNIP* and *ANXA1*, supporting an enhanced survival potential of these cells in Ns (Supplemental Figure 4, C and E). As a reference, analysis of tonsillar single cells showed differential expression of relevant genes among CD4^+^ T cell subsets (Supplemental Figure 4F). With respect to the potential CD4^+^ Treg transcriptomics profile, genes like *FOXP1*, a cofactor for FoxP3 (41), and *IRF1*, an inducer of type 1 regulatory Tr1 cells (42) and negative regulator of Tfh cell differentiation (43), were upregulated in NN compared with N participants (Figure 3D). Furthermore, downstream targets for several established Treg inducers including *STAT3*, *STAT5*, *STAT6* (44–47), *MYC* (48), *SMAD3* (49), vitamin D receptor (*VDR*) (50), and *FOXP3*/*FOXP1* were upregulated in NNs compared with Ns (Figure 3D). Our data indicate that, despite the significantly higher frequency of FOXP3^hi^ CD4^+^ T cells in Ns (Figure 3B), CD4^+^ T cells from NNs were characterized by a molecular profile favoring the development of potential suppressor CD4^+^ T cells. When we validated molecules revealed by scRNA-seq analysis with flow cytometry, we also observed significantly higher levels of combined TCF1^hi^AHR^hi^CAV1^hi^CD130^hi^ expression in Tfh cell subsets (PD-1^hi^CD57^lo/hi^) compared with naive CD4^+^ T cells (4.04% ± 3.65% vs. 0.21% ± 0.13%, *P* = 0.0261) and compared with the PD-1^lo^CD57^lo^ CD4^+^ T cell subset (4.04% ± 3.65% vs. 0.17% ± 0.17%, *P* = 0.0244) in tonsillar cell suspensions (Supplemental Figure 5A). In sum, our data suggest that Tfh cells from Ns expressed a molecular program that favored their differentiation and stemness.

### Cross-neutralization is associated with a lower cell density of IL-10^hi^CD25^hi^FoxP3^hi^ follicular regulatory CD4^+^ T cells.

Our transcriptomics analysis revealed a significantly higher frequency of FoxP3^hi^ cells in N compared with NN tissues (Figure 3B). We then used an in situ approach to quantify FoxP3^hi^ CD4^+^ T cells in extrafollicular and follicular LN areas. In line with our scRNA analysis (Figure 3B), we found a substantially (*P* = 0.1797) higher number of normalized FoxP3^hi^ CD4^+^ T cells within the follicular areas in Ns compared with NNs (Figure 3E, Supplemental Figure 3A, and Supplemental Figure 5B). We further investigated the FoxP3^hi^ CD4^+^ T cell compartment using CD25 (51) and IL-10, a cytokine that correlates with increased Treg functionality and suppressive capacity (52) (Supplemental Figure 6A). Analysis of CD25^hi^FoxP3^hi^ CD4^+^ T cells revealed comparable normalized numbers of extrafollicular CD25^hi^FoxP3^hi^ CD4^+^ T cells among the 2 categories (Figure 3F). However, there was a trend for lower numbers of follicular CD25^hi^FoxP3^hi^ CD4^+^ T cells (Tfr) and substantially lower cell density (*P* = 0.0823) of follicular IL-10^hi^CD25^hi^FoxP3^hi^ CD4^+^ T cells in the N tissues compared with the NN tissues (Figure 3, F and G). We found a significantly higher (*P* = 0.01691) ratio of FoxP3^hi^/Tfh CD4^+^ T cells in Ns compared with NNs, whereas the opposite was the case when we calculated the CD25^hi^FoxP3^hi^/Tfh CD4^+^ T cell ratio (data not shown). Our data suggest a GC-specific effect, rather than a global effect, of increased numbers of functional IL-10–producing Tfr cells in the follicles of NNs, which may contribute to limited GC Tfh cell responses.

### Cross-neutralization is associated with a higher degree of viral evolution.

**Figure 4:**
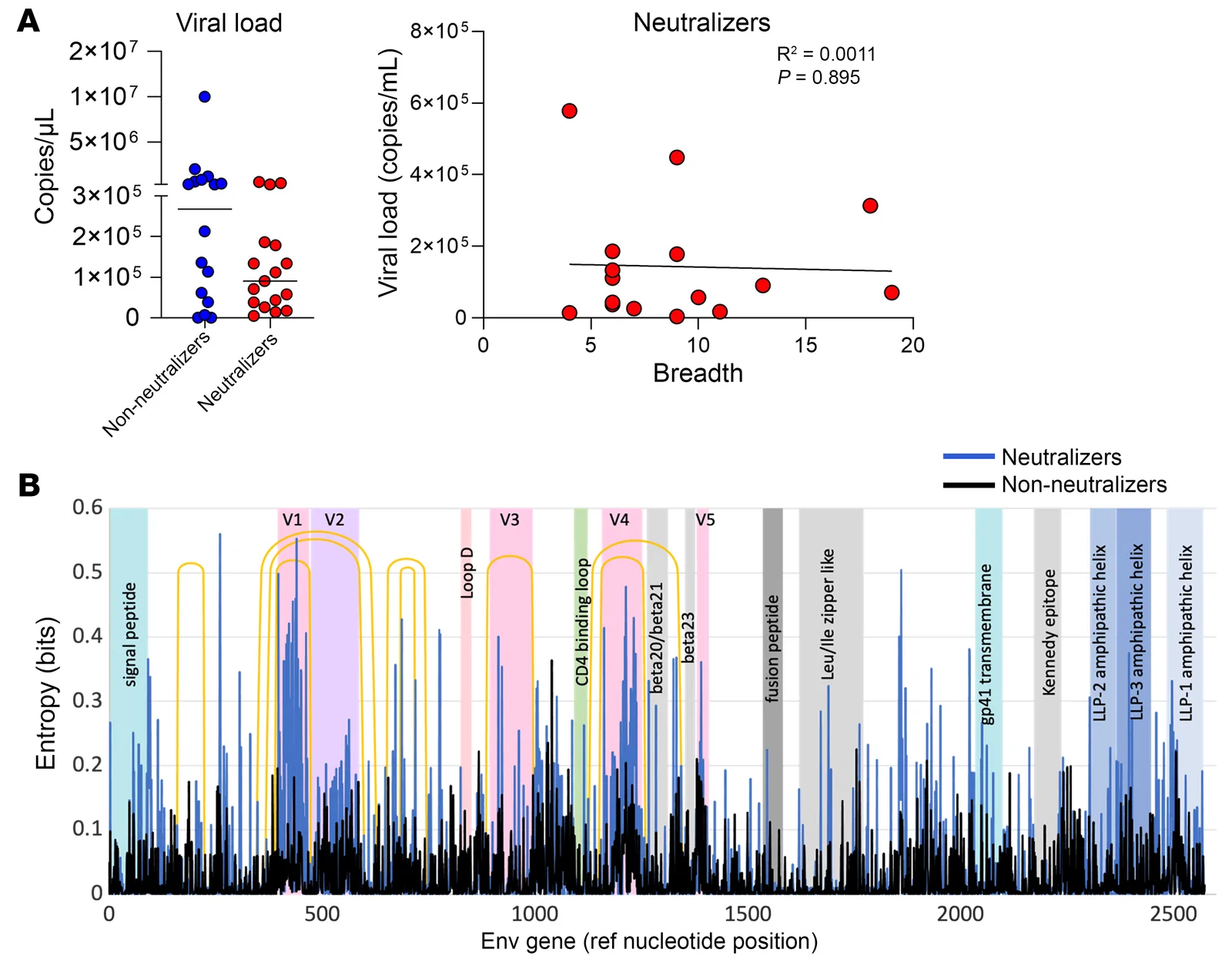
Higher viral evolution is associated with a lower in situ prevalence of actively transcribed virus in cross-neutralization participants. (**A**) pVLs in all study participants and association of VLs with neutralization breadth (number of isolates recognized out of the 20 isolates tested). Median values are shown. (**B**) Entropy-H(x) plot showing the location of positions with residue mismatches on alignment to the reference Env HIV sequence and the distribution of those mismatches over different HIV structural regions. Color shades denote distinct structural regions, and orange arcs the locations of gp120 variable loops.

To explore relationships between the aforementioned CD4^+^ T cell profiles and virus dynamics, we compared NN and N groups according to the plasma viral load (pVL), the prevalence of HIV RNA^+^ cells quantified by ISH, and the intra-host diversity of HIV *env* gene sequences. A trend, although not significant, for higher plasma viremia was found in NN LNs compared with N LNs (Figure 4A). The plasma viremia, however, did not correlate with the breadth of NAbs (Figure 4A). When the in situ expression of *HIV* mRNA was analyzed, we found significantly more *HIV* RNA^+^ cells (*P* = 0.0232) and a trend for higher levels of total viral RNA (*P* = 0.0704) in NN compared with N LNs (Supplemental Figure 6B). In line with the in situ analysis, more LN CD4^+^ T cells harboring *HIV* mRNA, analyzed by scRNA-seq, were detected in NNs compared with Ns (Supplemental Figure 6C). A strong association between the FDC area and RNA^+^ virions was found both in Ns and NNs (Supplemental Figure 6D). We detected a higher intra-host *env* diversity in Ns than in NNs, based on entropy analysis using high-throughput, single-genome amplification and sequencing (HT-SGS) (53) (Figure 4B and Supplemental Figure 7A). Intra-host diversity of *env* sequences in1 individual with a broad Ab neutralizing profile was among the highest entropy profiles (Supplemental Figure 7A). Of note, although this association could be consistent with positive selection by *env*-specific Abs in Ns, we also observed higher intra-host *env* sequence entropy in Ns when considering only synonymous variation (Supplemental Figure 7B). Moreover, several of the *env* gene regions with particularly high entropy in Ns did not correspond to known bNAb epitopes (Figure 4B). Taken together, these findings suggest higher cumulative levels of HIV evolution in Ns than in NNs.

### Cross-neutralizing activity is associated with a higher in situ prevalence of BC B cells expressing a molecular profile favoring GC development.

**Figure 5:**
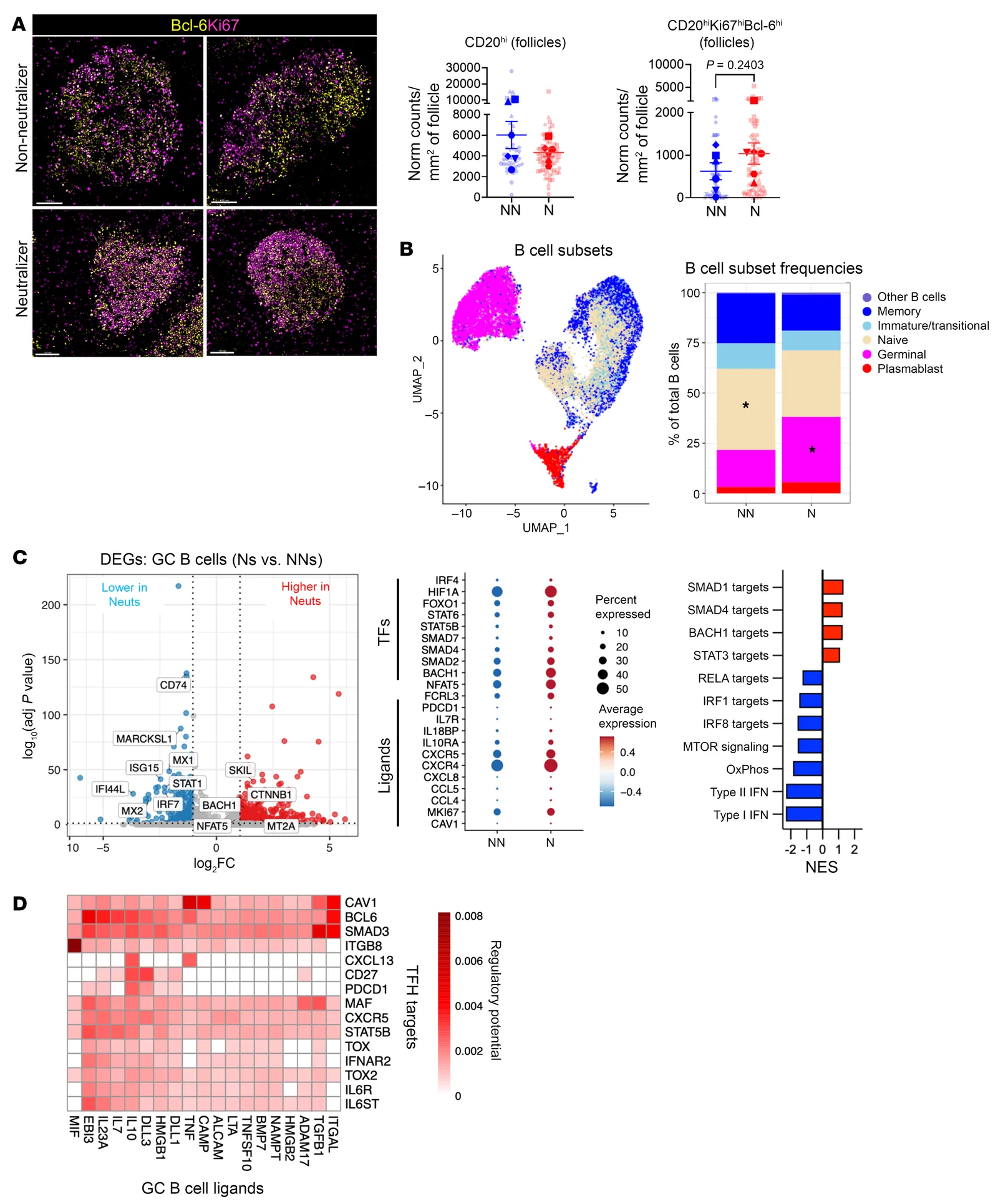
GC B cells express a molecular profile favoring GC development in cross-neutralization LNs. (**A**) Representative confocal images showing the distribution of BCL-6 (yellow) and Ki67 (magenta) within the GCs of NNs and Ns. Original magnification, ×40 with 1% zoom; scale bars: 100 μm. Superplots show normalized CD20^hi^ and Bcl-6^hi^Ki67^hi^CD20^hi^ B cell numbers of both individual (faint background symbols) and mean ± SEM (bold foreground symbols) follicular measurements in NNs (*n* = 6, blue) versus Ns (*n* = 6, red), as measured by quantitative imaging analysis and histocytometry. The different symbols represent different donors. For statistical comparisons using mean measurements, a Mann-Whitney *U* test was applied (*P* = 0.588, *P* = 0.2403). (**B**) UMAP projections of B cell populations identified by scRNA analysis in total l ymph node mononuclear cells, color-coded by B cell category. (**C**) Volcano plots and dot plots of DEGs and NESs from GSEA for selection of B cell–specific ligands and transcription factors. The DEGs were calculated using the MAST (Hurdle model–based) test via the FindMarkers function using the Seurat package in R. *P* values were corrected using the Benjamini-Hochberg method and genes meeting an adjusted *P < 0.05 and (log_2_FC) > 0.5 are highlighted on the volcano plot. Dots in the volcano plots represent genes that were significantly downregulated (blue) or upregulated (red) in Ns. (**D**) Heatmap of pairwise Tfh–GC B cell interactions as identified through ligand-target analysis. Darker colors denote interactions corresponding to an increased regulatory potential.

Finally, we sought to assess whether HIV neutralization could be linked to a specific GC B cell profile. To this end, we analyzed the numbers of GC B cells and total B cells (CD20^hi/dim^) in situ. Despite the comparable total numbers of B cells between the 2 groups, we found a trend for higher normalized numbers (*P* = 0.2403) of CD20^hi^Ki67^+^Bcl-6^hi^ B cells in LNs of Ns compared with NNs (Figure 5A and Supplemental Figure 8A). Furthermore, a significantly higher Tfh/B cell ratio (*P* < 0.0001) and a trend for higher Bcl-6^hi^ Tfh/Bcl-6^hi^Ki67^hi^ B cell ratios were found in Ns compared with NNs (Supplemental Figure 8B). We further performed scRNA-seq on B cells to elucidate their transcriptomic profile. Further clustering based on the expression of B cell–specific genes (Supplemental Figure 8C) revealed significantly higher GC B cell frequencies and lower naive B cell frequencies in N LNs than NN LNs (Figure 5B). This observation was further supported by the higher expression of *CXCR5* and *CXCR4* in N LNs than NN LNs (Figure 5C). Moreover, we found increased expression of factors promoting GC B cell differentiation (*BACH1*, *STAT6*), as well as the development of the dark zone (DZ) B cell compartment (*FOXO1*, *TGFB*/*SMAD2/4*) and the response to local osmotic stress (*NFAT5*) in N B cells compared with NN B cells (Figure 5C). NN B cells were found to upregulate type I and type II IFN and mTOR signaling, which could have a detrimental effect on GC B cell development (Figure 5C). Together, these data show that Ns were programmed for the development and maintenance of GC B cells. Furthermore, possible molecular targets that could mediate the interaction between GC B cells and Tfh cells were investigated across all 8 participants using the NicheNet package in R. Our analysis revealed novel putative “molecular pairs” that could mediate such interaction, including macrophage migration inhibitory factor (*MIF*) (ligand for CXCR4 and CD74 and potential regulator of B cells at trafficking, survival, and antigen presentation level) (54); integrin-β 8 (*ITGB8*) (unit of the avb8 integrin, a critical activator of TGF-β) (55); and *TGFB1/SMAD3* (Figure 5D). Therefore, our analysis reveals molecules that could serve as targets for in vivo interventions aiming to strengthen the Tfh–B cell interaction and bNAb development.

## Discussion

A subset of PLWH, independent of vaccination, can develop NAbs against HIV (56). Although the characteristics of a variety of bNAbs have been previous explored, the human follicular/GC immune dynamics that mediate the development of these Abs are largely unknown, mainly due to unavailability of relevant LNs from PLWH.

HIV is associated with a wide spectrum of lymphadenopathies, including follicular hyperplasia, lysis and involution (4, 57, 58). Our morphological analyses suggest that the follicular/GC areas of Ns are better preserved, providing the ground for more efficient development of GC immunoreactions. One could hypothesize that the preservation of follicular architecture, may reflect the in vivo function of homologous bNAbs rather than representing a driving force for their development. The development of NAbs in HIV or SHIV infection necessitates a long-term “synchronization” between the evolution of the Env protein and the maturation of Env-specific Tfh cell responses (58). Given the previous reports, one should not expect natural infection-induced bNAb activity during the acute/early post -infection stages. Indeed, bNAbs were detected 44–47 weeks after SHIV infection in NHPs (59–61). A similar time window for the development of anti-HIV bNAbs was found in PLWH (62). In our cohort, all PLWH participants were chronically infected. Presumably, the alterations in LN microarchitecture and cellularity most likely take place during the acute-to-early chronic phase of the infection. We argue that the preservation of follicular structures and associated cellular/molecular profiles preceded the appearance of bNAbs and guided their development. However, one limitation of our study is the overall young age of the PLWH studied. Whether the described profiles apply also to older individuals, in which aging can significantly alter the F/GC structure and cellularity, is not known and needs investigation using relevant LNs.

Differentiation of Tfh cells is a multiphase process driven by changes in gene expression due to intrinsic and surrounding microenvironmental cues that yield a cell pool with high heterogeneity (39). CD57^hi^PD1^hi^ human Tfh cells have an augmented capacity to secrete IL-4, at least in vitro (39), presumably representing Th2-like Tfh cells. While there was a significant expansion of Th1-like (CD57^lo^CXCR3^hi^) Tfh cells in the LNs of NNs, a cluster signature encompassing Th2-like (CD57^hi^CXCR3^lo^) Tfh cells appeared more prominently in Ns (63). Therefore, NAb development in PLWH could be associated with a significant expansion of Th2-like effector Tfh cells, in line with our previous data regarding the profile of SHIV Env–specific Tfh cells in NHPs with neutralizing anti-SHIV antibodies (59).

Tfh cells from N LNs were characterized by significant upregulation of genes and molecular pathways favoring their differentiation, function, and stemness, also supported by the significantly higher expression of *CD27*, a survival factor for memory CD4^+^ T cells and necessary for long-lived T cells (64), compared with NN participants. Tfh cells from NNs expressed signs of a less differentiated stage (high expression of *CD69*, *IL7R*, and *STAT6*, a profile found in tonsillar pre-Tfh cells) that could be mediated, at least in part, by the higher expression of *FOXP1*, a known negative regulator of Tfh cell differentiation (43) as well as by increased type I/II IFN signatures. Our data revealed increased type I and II IFN signaling signatures in both Tfh and B cells in NN participants. Type I IFN signaling could alter the differentiation of CD4^+^ T cells toward a Th1 phenotype (65), in line with the reduction in numbers of Bcl-6^hi^ Tfh cells found in NN participants. The significant upregulation of type I IFN molecular signatures in F/GCs from individuals with systemic lupus erythematosus is associated with perturbed differentiation of Tfh cells, especially the CD57^hi^ Tfh cell subset (66). Whether this is a generalized mechanism dysregulating the F/GC immune dynamics in chronic inflammatory diseases needs further investigation.

Tfr cells regulate Tfh function by limiting their numbers and curtailing their ability to provide B help through ICOS costimulation as well as IL-21 and IL-4 production (25, 51, 67). Since Tfr cells have been shown to expand both proportionally and numerically during HIV and SIV infection (25), we asked if differences in neutralization could also be due to differences in the frequencies of Treg and Tfr cell populations between the 2 study groups. We found substantially fewer Tfr cells in Ns, suggesting that Tfh cells in N PLWH were exposed to a less immunosuppressive F/GC microenvironment. On the other hand, Tregs exposed to local a inflammatory microenvironment adopt a strong suppressive function (68). The significant reduction in IL-10^hi^CD25^hi^FoxP3^hi^ Tfr cells found in N participants compared with NN participants further corroborates a less inflammatory follicular environment in these individuals.

LNs harbor a higher viral burden than is found in the peripheral blood in the asymptomatic phase of HIV infection (69–71). The FDC network is a stable “reservoir” of highly infectious virus (72, 73), supporting the maintenance of a viral reservoir (74, 75), possibly through a TNF-α mechanism (76), even after ART initiation (77). Although our data suggest that the FDC-mediated support of HIV transcription was operating in both NNs and Ns, this process alone cannot explain the higher levels of viral replication seen in the NNs. Previous studies have reported a modest positive association between HIV viral load (VL) and bNAb production (78), while others did not, especially for higher VLs (79). In our study, we did not observe a positive correlation between the pVL and neutralization breadth, suggesting that the viral burden per se (copies/μL) could not fully account for the neutralization activity seen in Ns, which is in line with observations from other cohorts (80). We detected a greater evolution of Env protein in Ns than in NNs, which corroborates previously published findings (59). It should be noted that the cross-sectional nature of our study did not allow us to determine when these *env* variants may have appeared. Circulating viruses in the blood usually represent a mix of viruses sensitive and resistant to the temporally coincident autologous bNAbs (81). In addition, most elite Ns, from whom several known bNAbs have been isolated, have detectable plasma viremia (82). In line with other studies in the field (83, 84), we found that donor neutralization activity mapped mainly to 1 or 2 specificities, which may not be sufficient to lower the contemporaneous virus levels in the plasma over the long term. As such, we cannot exclude the possibility that, at some point in infection, Ns may have harbored a higher VL. However, beyond the relative presence of actively transcribed virus, our data indicate an augmented evolution of the virus in Ns, critically contributing to the development of bNAbs (58).

The transcriptional profiling indicates a higher prevalence of DZ GC B cells and preservation of an anatomically distinct DZ (upregulation of *FOXO1*, *CXCR4*) in N compared with NN participants, in whom light zone (LZ) GC B cells may be more dominant (higher expression of *MTOR*) (85–88). It is well established that type I and type II IFNs are potent activators of the mTOR/PI3K pathway (89). During acute viral infections, an early boost of both type I and II IFN-mediated signaling followed by PI3K/AKT/mTOR pathway activation is beneficial for GC formation and for the generation of antigen-specific plasma cells (90–92). However, hyperactivation of the mTORC1 complex, fueled by sustained IFN signaling or other stimuli during chronic viral infection, restricts GC B cells in the DZ, which results in decreased access to antigen and Tfh cells in the LZ (91). Moreover, activation of mTORC1 can induce class-switch recombination in murine B cells, favoring IgG1-expressing B cell clones (92, 93). Considering that the IgG1 isotype of some bNAbs was reported to exhibit reduced neutralization capacity of viral-escape HIV variants compared with IgG3 and IgA1 (94), we propose that sustained mTORc1 signaling might be a limiting factor for the generation of potent neutralizing Abs. Together, our study suggests that the development of NAbs during chronic HIV infection is multifactorial and associated with (a) a “controlled” development and maturation of follicular structures; (b) the in situ operation of molecular signals favoring the interaction between Tfh cells (“help”) and B cells (activation, maturation); and (c) a higher viral evolution, in line with our previous data (59). Viral infections, autoimmunity (66), and aging (95) could have a dramatic effect on these factors, ultimately impairing the efficacy of vaccine-induced responses. We have recently shown that restoration of vaccine-induced Abs in aged SIV-infected NHPs subjected to combined ART was associated with downregulation of Tfh and B cell IFN signaling pathways (96). Newer strategies that build on GC biology and dynamics such as (a) germline-targeting vaccines (97) and (b) novel immunogens targeting FDCs and a prolonged presence of the immunogen within the GC area (98) are yielding promising results in this regard in preclinical and clinical settings. Furthermore, establishing circulating biomarkers and surrogates of the operation of such signals within the LNs, especially the GCs, would be highly informative for the monitoring of vaccine-induced Ab responses.

One limitation in our study is the small number of participants with bNAbs that was analyzed, which mirrors the small percentage of individuals with extremely broad and potent bNAb activity in clinical cohorts (~1%) (99, 100), and the rare occurrence of PLWH who remain ART naive. Given these limitations, confirmation of our findings in a second, validation cohort was not possible in the present study. To overcome this limitation, we performed parallel experiments using multiple platforms and observed correlative relationships across and within our small groups, which we believe strengthens our reported observations. A second limitation in our study is that the exact timing of participant HIV acquisition could not be ascertained. As such, differences in the approximated lengths of viral infection could exist. Even with this limitation, however, the requirement for a better-preserved follicular microarchitecture remains. Our analysis also elucidates B and T cell molecules that could represent novel targets for interventions aiming to strength the Tfh–B cell interaction and Ab responses against pathogens or immunogens.

## Methods

### Sex as a biological variable.

Details of the sex of the participants in the study are reported in Supplemental Table 1 (Clinical Information). Sex as a biological variable was not investigated in our study, since 30 of 33 participants reported being male. The sex of the participants mirrors the sex distribution in the clinical cohort from which the participants were drawn. Future work is needed to extend our findings in women.

Additional methods can be found in the Supplemental Methods.

### pVL and CD4^+^ T cell counts.

Plasma samples, LN cell suspensions, and biopsy samples were obtained from untreated, chronically infected PLWH, with explicit written informed consent from the participants prior to donation. None of the participants included in the protocol had active opportunistic infections, and all were HBV and HCV negative. LN biopsies were obtained from PLWH who had a palpable LN in the cervical area or a LN detected by ultrasound in the inguinal area. The pVL was quantified by automated real-time PCR using the m2000 system (Abbott). The range of detection for pVL was 40–10,000,000 copies/mL. CD4^+^ T cell counts were determined by flow cytometry using the TruCount kit with the FACSCanto II instrument (BD Bioscience), according to the manufacturer’s instructions. Tonsillar and LN cell suspensions were stored at liquid nitrogen until further use.

### Flow cytometric data acquisition and analysis.

Surface and intracellular staining of LN- and tonsil-derived cells was carried out using the appropriate titrated Abs (Supplemental Table 3). Events were collected on a BD X50 Symphony (BD Biosciences), and electronic compensation was performed with Ab capture beads (BD Biosciences). Data were analyzed using FlowJo, version 10.8.1 (TreeStar, BD). Dimensionality reduction analysis was performed using the *t*-SNE and FlowSOM plugins on FlowJo (Tfh cell populations). For *t*-SNE analysis of Tfh cells, individual cell populations were manually gated in FlowJo, and fcs files were exported for each participant. Files were preprocessed to exclude batch-to-batch variations and concatenated to a single file downsampled to 11,481 cells/sample, yielding a file suitable for *t*-SNE analysis. *t*-SNE parameters were set to 1,000 iterations, learning rate: 2,818 and perplexity 30. Clustering analysis using the FlowSOM plugin (FlowJo) was performed for the following 11 markers: T cell immunoreceptor with Ig and ITIM domains (TIGIT), ICOS, CD95, CD57, CXCR3, OX40, cytotoxic T-lymphocyte associated protein 4 (CTLA-4), programmed death ligand-1 (PDL-1), CD226, OX40L, T-cell immunoglobulin and mucin-domain containing-3 (TIM-3), for a grid size of 10 × 10, yielding 8 meta-clusters. For visualization, clustering results were overlayed on the *t*-SNE projection using the ClusterExplorer plugin. Differences in frequencies in FlowSOM-generated clusters were calculated using an unpaired *t* test in GraphPad Prism (version 9.3.1).

### Multiparameter confocal imaging-data acquisition and analysis.

Unconjugated primary and secondary and conjugated Abs were used for the staining of paraformaldehyde-fixed, paraffin-embedded tissue sections (Supplemental Table 4). For viral RNA visualization, RNAscope ISH was performed according to the manufacturer’s instructions for formalin-fixed, paraffin-embedded tissues using dedicated HIV RNA probes (catalog 416111, Advanced Cell Diagnostics) and the RNAscope Multiplex Fluorescent Reagent Kit v2 (Advanced Cell Diagnostics). The assay was performed as previously reported (101) but without proteinase K treatment in order to preserve the human CD20 epitope. Confocal images were obtained on a Leica TCS SP8 confocal system, at 512 × 512 pixel density and ×1 optical zoom using a ×40 objective (NA 1.3), unless otherwise stated. No frame averaging or summing was used while obtaining the images. To ensure accurate representation and minimize selection bias, at least 50% of the tissue was imaged. Fluorophore spillover, when present, was corrected by imaging tissues stained with single Ab-fluorophore combinations, and by creating a compensation matrix via the Leica LAS-AF Channel Dye Separation module (Leica Microsystems) according to the user’s manual. Confocal images were analyzed with ImageJ software (102) and Imaris, version 9.5.0 (Bitplane). Histocytometric analysis was performed to generate quantitative data from the images, as previously described (32, 39, 101). Fragmented follicles, follicles partially on edge, and follicles with indeterminate boundaries were excluded from the analysis. To define FDCs, we used the mAb CNA.42, which recognizes a 120 kDa formalin-resistant, evolutionarily conserved glycosylated antigen that is mainly expressed on FDCs (33). In LNs, FDCs form intricate interdigitating networks on the surface of which antigen is trapped (103). To define these networks, we performed network area surface calculation using the Imaris software (Bitplane) surface creation module. The number of FDC-bound virions in RNAscope analysis was also measured using Imaris software. Quantitation was performed using the Spots creation feature after appropriately masking the vRNA^+^ channel to reveal colocalization with FDC surfaces. For RNA virion quantification, a thresholding diameter of 2 μm was used in a manner consistent with previous work in our laboratory (104).

### scRNA-seq data analysis.

FASTQ files were uploaded to Cell Ranger on the 10X Genomics cloud, and no depth normalization was carried out. The filtered count matrix was then analyzed using the Seurat package in R. Cell annotation was carried out using SingleR, and the reference expression dataset was derived from the MonacoImmuneData atlas from the celldex R package. Doublet cells were removed from the analysis using the DoubletFinder package in R. Differentiation gene expression was assessed using the MAST R package. Clustered cells were visualized using uniform manifold approximation and projection (UMAP), and global differences between clusters were assessed using principal component analysis (PCA).

### HT-SGS of HIV env.

Plasma samples were thawed at room temperature, followed by a brief centrifugation to collect liquid from the sides of the tubes. RNA extraction from virions in the supernatant was conducted using the QIAamp Viral RNA Mini Kit (Qiagen, 52906), following the instructions provided by the manufacturer. Subsequent processing steps were similar to those described in our previous study (53).

### Statistics.

We compared differences between groups (controls, NNs, and Ns) in flow cytometry and histocytometry using nonparametric, unpaired Mann-Whitney *U* and nonparametric 1-way ANOVA statistical tests (Kruskal-Wallis followed by Dunn′s multiple-comparison test). A parametric *t* test was used for the *t*-SNE cluster analysis, whereas data with more than 1 explanatory variable were analyzed using 2-way ANOVA followed by Tukey’s post hoc comparisons. In those cases where the dependent variable was not normally distributed according to the Lilliefors and/or Shapiro-Wilk normality tests, a box-cox transformation was applied, and when the transformation did not render a normally distributed variable, the nonparametric Mann-Whitney *U* test for unpaired variables was used. GraphPad Prism (version 10.2.1), JASP (version 0.18.3), and the program R studio (version 2023.06.2+561 with the packages nortest [10.1-4] and ggplot2 [3.5.0]) were used to create the graphs and perform the *t* tests, Mann-Whitney *U* tests, and ANOVA tests, respectively. All quantitative data show the ± SD, except for the box plots, which show IQRs. Graphs comprising multiple ROIs (follicles) were visualized as SuperPlots, depicting both individual measurements and their corresponding mean ± SEM. Statistical analyses were conducted on the mean measurements only. *P* values and probability values of less than 0.05 were considered statistically significant.

### Study approval.

All samples from PLWH were procured with explicit written informed consent from participants prior to donation, adhering strictly to the principles outlined in the Declaration of Helsinki. The utilization of these samples was formally sanctioned by both the research committee and the ethics in research committee of the National Institute of Respiratory Diseases “Ismael Cosío Villegas,” Mexico City as part of the “C71-18” protocol. The control tissue samples were retrieved from the archives of the Institute of Pathology of Lausanne University Hospital, Switzerland, and their use was approved by the ethics committee of the Canton de Vaud, Switzerland (protocol number 2021-01161). Anonymized, discarded pathologic tonsil specimens were obtained from Children’s National Medical Center (CNMC) under the auspices of the Basic Science Core of the District of Columbia Developmental Center for AIDS Research. The CNMC IRB determined that the study of anonymized discarded tissues did not constitute “human subjects research.” Sample sizes were not predetermined by power calculations, and investigators were not blinded to group identity during the study.

### Data availability.

Supporting data values are available in the Supporting Data Values file. The authors agree to share all publication-related data. For further information, contact the corresponding author. The scRNA-Seq data discussed in this publication were deposited in the Gene Expression Omnibus (GEO) database (GSE288212).

## Author contributions

EM and AAS performed experiments, analyzed and interpreted data and drafted the manuscript. SO, SG, PMDRE, MHB, SHK, FB, MO, and CMB performed experiments. ABE, GK, and AA participated in scRNA analysis and drafted the manuscript. NDR and EAB analyzed and interpreted data and drafted the manuscript. FTR, SAR, GRT, YALV, MGN, and LDL provided participant material and clinical data. HL performed scRNA/HIVmRNA and tonsillar scRNA analysis. RG, SM, JRM, RPS, and RAK provided supervision, interpreted the data, and critically revised the manuscript. CP conceived, designed and supervised the study, interpreted data, and revised the manuscript. All authors have read and approved the final version for submission.

## Conflict of interest

RG has received consulting income from Takeda and Sanofi and declares ownership in Ozette Technologies and Modulus Therapeutics.

## Funding support

This work is the result of NIH funding, in part, and is subject to the NIH Public Access Policy. Through its acceptance, NIH has been given the right to make the work publicly available in PubMed Central.

- Intramural Research Program of the Vaccine Research Center, NIAID, NIH.
- Swiss National Science Foundation (SNF, 310030_204226, to CP).
- NIAID, NIH (UM1 AI164561, to CP, AAS, and RS).

## Supplementary Material

Supplemental data

Supporting data values

## Figures and Tables

**Table 1:**
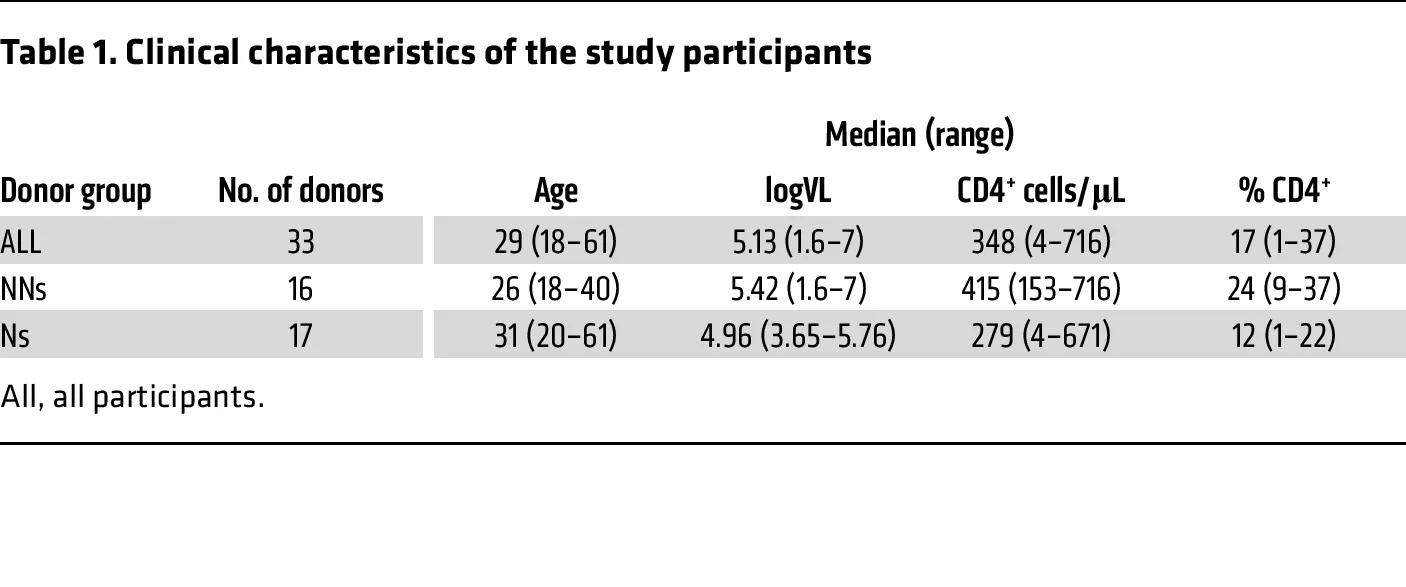
Clinical characteristics of the study participants

## Version 1 07/14/2026

In-Press Preview

## Version 2 09/01/2026

Electronic publication

## References

[1] Estes JD (2013). Pathobiology of HIV/SIV-associated changes in secondary lymphoid tissues. Immunol Rev.

[2] Zeng M (2012). Lymphoid tissue damage in HIV-1 infection depletes naive T cells and limits T cell reconstitution after antiretroviral therapy. PLoS Pathog.

[3] Grouard G, Clark EA (1997). Role of dendritic and follicular dendritic cells in HIV infection and pathogenesis. Curr Opin Immunol.

[4] Paiva DD (1996). Spectrum of morphologic changes of lymph nodes in HIV infection. Mem Inst Oswaldo Cruz.

[5] Cubas RA (2013). Inadequate T follicular cell help impairs B cell immunity during HIV infection. Nat Med.

[6] Moir S, Fauci AS (2017). B-cell responses to HIV infection. Immunol Rev.

[7] Niessl J (2020). Persistent expansion and Th1-like skewing of HIV-specific circulating T follicular helper cells during antiretroviral therapy. EBioMedicine.

[8] Pallikkuth S (2017). T follicular helper cells and B cell dysfunction in aging and HIV-1 infection. Front Immunol.

[9] Breitfeld D (2000). Follicular B helper T cells express CXC chemokine receptor 5, localize to B cell follicles, and support immunoglobulin production. J Exp Med.

[10] Johnston RJ (2009). Bcl6 and Blimp-1 are reciprocal and antagonistic regulators of T follicular helper cell differentiation. Science.

[11] Shlomchik MJ, Weisel F (2012). Germinal center selection and the development of memory B and plasma cells. Immunol Rev.

[12] Hong JJ (2014). Early lymphoid responses and germinal center formation correlate with lower viral load set points and better prognosis of simian immunodeficiency virus infection. J Immunol.

[13] Petrovas C (2012). CD4^+^ T follicular helper cell dynamics during SIV infection. J Clin Invest.

[14] Lindqvist M (2012). Expansion of HIV-specific T follicular helper cells in chronic HIV infection. J Clin Invest.

[15] Chakhtoura M (2021). Germinal center T follicular helper (GC-Tfh) cell impairment in chronic HIV infection involves c-Maf signaling. PLoS Pathog.

[16] Caskey M (2020). Broadly neutralizing antibodies for the treatment and prevention of HIV infection. Curr Opin HIV AIDS.

[17] McCoy LE, McKnight A (2017). Lessons learned from humoral responses of HIV patients. Curr Opin HIV AIDS.

[18] Klein F (2013). Somatic mutations of the immunoglobulin framework are generally required for broad and potent HIV-1 neutralization. Cell.

[19] Scheid JF (2009). Broad diversity of neutralizing antibodies isolated from memory B cells in HIV-infected individuals. Nature.

[20] Victora GD, Mouquet H (2018). What are the primary limitations in B-cell affinity maturation, and how much affinity maturation can we drive with vaccination? Lessons from the antibody response to HIV-1. Cold Spring Harb Perspect Biol.

[21] Locci M (2013). Human circulating PD-1^+^CXCR3^–^CXCR5^+^ memory Tfh cells are highly functional and correlate with broadly neutralizing HIV antibody responses. Immunity.

[22] Cohen K (2014). Early preservation of CXCR5^+^ PD-1^+^ helper T cells and B cell activation predict the breadth of neutralizing antibody responses in chronic HIV-1 infection. J Virol.

[23] Moody MA (2016). Immune perturbations in HIV-1-infected individuals who make broadly neutralizing antibodies. Sci Immunol.

[24] Townsley SM (2021). B cell engagement with HIV-1 founder virus envelope predicts development of broadly neutralizing antibodies. Cell Host Microbe.

[25] Miles B (2015). Follicular regulatory T cells impair follicular T helper cells in HIV and SIV infection. Nat Commun.

[26] Sarzotti-Kelsoe M (2014). Optimization and validation of the TZM-bl assay for standardized assessments of neutralizing antibodies against HIV-1. J Immunol Methods.

[27] Wibmer CK (2015). HIV broadly neutralizing antibody targets. Curr Opin HIV AIDS.

[28] Raju N (2019). NFPws: a web server for delineating broadly neutralizing antibody specificities from serum HIV-1 neutralization data. Bioinformatics.

[29] Doria-Rose NA (2017). Mapping polyclonal HIV-1 antibody responses via next-generation neutralization fingerprinting. PLoS Pathog.

[30] Schacker TW (2002). Collagen deposition in HIV-1 infected lymphatic tissues and T cell homeostasis. J Clin Invest.

[31] Zannini C (2000). A video densitometric analysis of viral burden and follicular dendritic cell damage in lymph nodes in the latency phase of HIV infection. Cytometry.

[32] Moysi E (2018). Altered immune cell follicular dynamics in HIV infection following influenza vaccination. J Clin Invest.

[33] Raymond IAS (1997). CNA.42, a new monoclonal antibody directed against a fixative-resistant antigen of follicular dendritic reticulum cells. Am J Pathol.

[34] Sun X, Kaufman PD (2018). Ki-67: more than a proliferation marker. Chromosoma.

[35] Legendre C (1998). CD80 expression is decreased in hyperplastic lymph nodes of HIV^+^ patients. Int Immunol.

[36] Yasutomi M (2022). CD226 and TIGIT cooperate in the differentiation and maturation of human Tfh Cells. Front Immunol.

[37] Choi YS (2011). ICOS receptor instructs T follicular helper cell versus effector cell differentiation via induction of the transcriptional repressor Bcl6. Immunity.

[38] Tahiliani V (2017). OX40 cooperates with ICOS to amplify follicular Th cell development and germinal center reactions during infection. J Immunol.

[39] Padhan K (2021). Acquisition of optimal Tfh cell function is defined by specific molecular, positional, and TCR dynamic signatures. Proc Natl Acad Sci U S A.

[40] Kim JR (2005). Human CD57^+^ germinal center-T cells are the major helpers for GC-B cells and induce class switch recombination. BMC Immunol.

[41] Konopacki C (2019). Transcription factor Foxp1 regulates Foxp3 chromatin binding and coordinates regulatory T cell function. Nat Immunol.

[42] Giang S, La Cava A (2017). IRF1 and BATF: key drivers of type 1 regulatory T-cell differentiation. Cell Mol Immunol.

[43] Wang H (2014). The transcription factor Foxp1 is a critical negative regulator of the differentiation of follicular helper T cells. Nat Immunol.

[44] Pallandre JR (2007). Role of STAT3 in CD4^+^CD25^+^FOXP3^+^ regulatory lymphocyte generation: implications in graft-versus-host disease and antitumor immunity. J Immunol.

[45] Chaudhry A (2009). CD4^+^ regulatory T cells control TH17 responses in a Stat3-dependent manner. Science.

[46] Betts BC (2014). STAT5 polarization promotes iTregs and suppresses human T-cell alloresponses while preserving CTL capacity. J Leukoc Biol.

[47] Chu KH (2020). STAT6 pathway is critical for the induction and function of regulatory T cells induced by mucosal B cells. Front Immunol.

[48] Saravia J (2020). Homeostasis and transitional activation of regulatory T cells require c-Myc. Sci Adv.

[49] Jana S (2009). The role of NF-kappaB and Smad3 in TGF-beta-mediated Foxp3 expression. Eur J Immunol.

[50] Fisher SA (2019). The role of vitamin D in increasing circulating T regulatory cell numbers and modulating T regulatory cell phenotypes in patients with inflammatory disease or in healthy volunteers: a systematic review. PLoS One.

[51] Chung Y (2011). Follicular regulatory T cells expressing Foxp3 and Bcl-6 suppress germinal center reactions. Nat Med.

[52] Laidlaw BJ (2017). Interleukin-10 from CD4^+^ follicular regulatory T cells promotes the germinal center response. Sci Immunol.

[53] Ko SH (2021). High-throughput, single-copy sequencing reveals SARS-CoV-2 spike variants coincident with mounting humoral immunity during acute COVID-19. PLoS Pathog.

[54] Klasen C (2014). MIF promotes B cell chemotaxis through the receptors CXCR4 and CD74 and ZAP-70 signaling. J Immunol.

[55] Worthington JJ (2015). Integrin αvβ8-mediated TGF-β activation by effector regulatory T cells is essential for suppression of T-cell-mediated inflammation. Immunity.

[56] Stamatatos L (2009). Neutralizing antibodies generated during natural HIV-1 infection: good news for an HIV-1 vaccine?. Nat Med.

[57] Chadburn A (2013). Lymphoid proliferations associated with human immunodeficiency virus infection. Arch Pathol Lab Med.

[58] Caponetti G, Pantanowitz L (2008). HIV-associated lymphadenopathy. Ear Nose Throat J.

[59] Yamamoto T (2015). Quality and quantity of Tfh cells are critical for broad antibody development in SHIVAD8 infection. Sci Transl Med.

[60] Shingai M (2012). Most rhesus macaques infected with the CCR5-tropic SHIV(AD8) generate cross-reactive antibodies that neutralize multiple HIV-1 strains. Proc Natl Acad Sci U S A.

[61] Walker LM (2011). Rapid development of glycan-specific, broad, and potent anti-HIV-1 gp120 neutralizing antibodies in an R5 SIV/HIV chimeric virus infected macaque. Proc Natl Acad Sci U S A.

[62] Doria-Rose NA (2014). Developmental pathway for potent V1V2-directed HIV-neutralizing antibodies. Nature.

[63] Austin JW (2019). Overexpression of T-bet in HIV infection is associated with accumulation of B cells outside germinal centers and poor affinity maturation. Sci Transl Med.

[64] Hendriks J (2000). CD27 is required for generation and long-term maintenance of T cell immunity. Nat Immunol.

[65] Ray J (2014). Transcription factor STAT3 and type I interferons are corepressive insulators for differentiation of follicular helper and T helper 1 cells. Immunity.

[66] Georgakis S (2025). Cellular and molecular determinants mediating the dysregulated germinal center immune dynamics in systemic lupus erythematosus. Front Immunol.

[67] Linterman MA (2011). Foxp3^+^ follicular regulatory T cells control the germinal center response. Nat Med.

[68] van der Veeken J (2016). Memory of inflammation in regulatory T cells. Cell.

[69] Pantaleo G (1993). HIV infection is active and progressive in lymphoid tissue during the clinically latent stage of disease. Nature.

[70] Tenner-Racz K (1998). The unenlarged lymph nodes of HIV-1-infected, asymptomatic patients with high CD4^+^ T cell counts are sites for virus replication and CD4^+^ T cell proliferation: the impact of highly active antiretroviral therapy. J Exp Med.

[71] Pantaleo G (1991). Lymphoid organs function as major reservoirs for human immunodeficiency virus. Proc Natl Acad Sci U S A.

[72] Spiegel H (1992). Follicular dendritic cells are a major reservoir for human immunodeficiency virus type 1 in lymphoid tissues facilitating infection of CD4^+^ T-helper cells. Am J Pathol.

[73] Heath S (1995). Follicular dendritic cells and human immunodeficiency virus infectivity. Nature.

[74] Schacker T (2000). Rapid accumulation of human immunodeficiency virus (HIV) in lymphatic tissue reservoirs during acute and early HIV infection: implications for timing of antiretroviral therapy. J Infect Dis.

[75] Pantaleo G (1998). Evolutionary pattern of human immunodeficiency virus (HIV) replication and distribution in lymph nodes following primary infection: implications for antiviral therapy. Nat Med.

[76] Thacker TC (2009). Follicular dendritic cells and human immunodeficiency virus type 1 transcription in CD4^+^ T cells. J Virol.

[77] Fletcher CV (2014). Persistent HIV-1 replication is associated with lower antiretroviral drug concentrations in lymphatic tissues. Proc Natl Acad Sci U S A.

[78] Doria-Rose NA (2010). Breadth of human immunodeficiency virus-specific neutralizing activity in sera: clustering analysis and association with clinical variables. J Virol.

[79] Sajadi MM (2011). Correlation between circulating HIV-1 RNA and broad HIV-1 neutralizing antibody activity. J Acquir Immune Defic Syndr.

[80] Moore PL (2015). Virological features associated with the development of broadly neutralizing antibodies to HIV-1. Trends Microbiol.

[81] Freund NT (2017). Coexistence of potent HIV-1 broadly neutralizing antibodies and antibody-sensitive viruses in a viremic controller. Sci Transl Med.

[82] Woldemeskel BA (2020). Viral reservoirs in elite controllers of HIV-1 infection: implications for HIV cure strategies. EBioMedicine.

[83] Walker LM (2010). A limited number of antibody specificities mediate broad and potent serum neutralization in selected HIV-1 infected individuals. PLoS Pathog.

[84] Bonsignori M (2012). Two distinct broadly neutralizing antibody specificities of different clonal lineages in a single HIV-1-infected donor: implications for vaccine design. J Virol.

[85] Albright A (2019). TGFβ signaling in germinal center B cells promotes the transition from light zone to dark zone. J Exp Med.

[86] Inoue T (2017). The transcription factor Foxo1 controls germinal center B cell proliferation in response to T cell help. J Exp Med.

[87] Dominguez-Sola D (2015). The FOXO1 transcription factor instructs the germinal center dark zone program. Immunity.

[88] Victora G, Nussenzweig M (2022). Germinal centers. Annu Rev Immunol.

[89] Platanias L (2005). Mechanisms of type-I- and type-II-interferon-mediated signalling. Nat Rev Immunol.

[90] Raybuck A (2018). B cell-intrinsic mTORC1 promotes germinal center-defining transcription factor gene expression, somatic hypermutation, and memory B cell generation in humoral immunity. J Immunol.

[91] Ersching J (2017). Germinal center selection and affinity maturation require dynamic regulation of mTORC1 kinase. Immunity.

[92] Chiu H (2019). The mTORC1/4E-BP/eIF4E axis promotes antibody class switching in B lymphocytes. J Immunol.

[93] Dahlgren M (2022). Type I interferons promote germinal centers through B cell intrinsic signaling and dendritic cell dependent Th1 and Tfh cell lineages. Front Immunol.

[94] Scheepers C (2020). Antibody isotype switching as a mechanism to counter HIV neutralization escape. Cell Rep.

[95] Moysi E (2022). Human lymph node immune dynamics as driver of vaccine efficacy: an understudied aspect of immune responses. Expert Rev Vaccines.

[96] Pallikkuth S (2023). IL-21-IgFc immunotherapy alters transcriptional landscape of lymph node cells leading to enhanced flu vaccine response in aging and SIV infection. Aging Cell.

[97] Sutton HJ (2025). Simultaneous induction of multiple classes of broadly neutralizing antibody precursors by combination germline-targeting immunization in nonhuman primates. Sci Immunol.

[98] Tokatlian T (2019). Innate immune recognition of glycans targets HIV nanoparticle immunogens to germinal centers. Science.

[99] Landais E, Moore PL (2018). Development of broadly neutralizing antibodies in HIV-1 infected elite neutralizers. Retrovirology.

[100] Sather DC S (2012). Broadly neutralizing antibodies developed by an HIV-positive elite neutralizer exact a replication fitness cost on the contemporaneous virus. J Virol.

[101] Moysi E (2021). In situ characterization of human lymphoid tissue immune cells by multispectral confocal imaging and quantitative image analysis; implications for HIV reservoir characterization. Front Immunol.

[102] Schneider CA (2012). NIH Image to ImageJ: 25 years of image analysis. Nat Methods.

[103] Allen CDC, Cyster JG (2008). Follicular dendritic cell networks of primary follicles and germinal centers: phenotype and function. Semin Immunol.

[104] Dias J (2021). Concordance of immunological events between intrarectal and intravenous SHIVAD8-EO infection when assessed by Fiebig-equivalent staging. J Clin Invest.

