# Supplementary material for: A favorable follicular helper CD4+ T cell programming characterizes neutralization activity in chronic HIV infection
Source: J Clin Invest. 2026 Jul 14;136(17):e192575. doi: 10.1172/JCI192575 (PMC13528940; doi:10.1172/JCI192575)
Supplement: Supplementary file 1 [file jci-136-192575-s031.pdf]

Supplemental Materials and Methods

Sample Collection and Processing

Upon receipt of tissue, lymph nodes and tonsils were cleaned with saline solution and placed in ice-cold RPMI 1640 media (GIBCO) supplemented with 10% Fetal Bovine Serum (Hyclone-Cytiva, USA), 2 mM L-glutamine, 100 U/mL penicillin and 100 μg/mL streptomycin (Invitrogen) and immediately transferred to the laboratory for further processing. All LN biopsy tissue samples were cleaned of surrounding fatty tissue and each cut in three small pieces: the first was placed in saline solution and used in microbiological studies to rule out bacterial, fungal and *Mycobacterium tuberculosis* presence (GenXpert assay, Cepheid Inc); the second, was placed in freshly prepared paraformaldehyde 4% for 24h and then transferred to Ethanol (EtOH) 80% followed by paraffin inclusion and used for histopathological examination to rule out neoplasms; and the third part was used for cell dissociation to be used in experiments of this study. For cell dissociation, lymphoid tissue was cut in small pieces with a scalpel and manually dissociated using a 70mm mesh and a syringe embolus. The cells obtained in suspension were centrifuged (1500 rpm, 10 minutes). Supernatant was discarded and the cell pellet was resuspended in complete media. Cells were counted and cryopreserved at a concentration of 10 million cells per milliliter in freezing medium (FBS supplemented with 10% DMSO, Sigma-Aldrich) and kept in liquid nitrogen until further use. The number of cells obtained varied between 1 and 45 million, depending on the size and chronicity of the infection. Microbiological tests and histopathological studies were conducted in INER laboratories, with results recorded in the participants' medical records. Additionally, peripheral blood was collected for plasma viral load determination and T lymphocyte counts at the virological diagnosis laboratory and the cytometry laboratory of CIENI. None of the participants of this study had opportunistic infections, and all were HBV and HCV negative. A similar protocol

was used for tonsil FFPE preparation, only tissues were placed in 10% Neutral Buffered Formalin for 24hrs before being embedded in paraffin and sectioned for multiplex confocal microscopy analysis. Tonsil LNMC were prepared in the same way as LN LNMCs.

Sample shipment

Vials containing 15–20 million cryopreserved cells were shipped in a liquid nitrogen container, with continuous temperature monitoring throughout transit. Upon thawing, viability was between 70-99%.

Flow cytometry

Lymph node mononuclear cells (1-2x10^6 cells/mL) from HIV+ lymph nodes and HIV- tonsils were thawed and rested for 1-hour at 37ºC. Cells were incubated with the Blue or Aqua LIVE/DEAD Fixable Dead Cell Stain Dye (Invitrogen), and surface-stained with titrated amounts of fluorophore-conjugated antibodies against: CD4, CD27, CD45RO, PD-1, CXCR5, TIGIT, CD95, CXCR3, ICOS, PDL-1, CD226, CD57, OX40, CTLA-4, OX40L, TIM3, CD8, CD19, CD130 for T cell phenotyping (**Supplemental Table 3**). For intracellular staining, cells were washed with PBS, fixed, permeabilized using the BD Transcription Factor Buffer Set (BD Biosciences), and stained with titrated amounts of fluorophore-conjugated antibodies against: AHR, CAV-1 and TCF1. Cells were then washed and fixed with 2% paraformaldehyde.

Multiparameter Confocal Imaging

Paraffin tissue sections 7-10 micron thick were deparaffinized and rehydrated through sequential Xylene-Ethanol-diH_20 solutions. Heat Induced Epitope Retrieval was performed using the Borg Decloaker reagent (Biocare Medical) for 15 minutes at 110ºC in a chamber pressurized at 5-6 PSI. This was followed by a blocking and permeabilization step using a Phosphate Buffered (PBS)/ Bovine Serum Albumin (BSA)/ Triton-X solution, after which tissue sections were stained

overnight at 4ºC with titrated amounts of primary antibodies. The next day, tissue sections were washed with PBS, and stained with secondary fluorophore-conjugated antibodies, followed by conjugated antibodies **(Supplemental Table 4)**. At the end of all staining rounds, a final washing step with PBS was performed, and nuclear stain, either JOJO-1 or JOPRO-1, was applied (ThermoFisher Scientific). Slides were mounted with a glass coverslip using Fluoromount G (Southern Biotech). For RNAscope assays, sections were baked for 1 hour at 60ºC and deparaffinized using serial xylene and ethanol baths. This was followed by an antigen retrieval step at 110ºC for 15 minutes. Subsequently, sections were incubated with the HIV-1 Clade B-specific probes at 40ºC for 2 hours in a HybEz hybridization oven (ACD) before being subjected to 4 rounds of signal amplification performed using the RNAscope amplification reagents. To visualize the RNA signal, a tyramide-based detection system was used (TSA Plus Cyanine 5, Akoya Biosciences) per manufacturer's instructions. All additional immunofluorescent stainings, as applicable, were performed at the end of the RNAscope protocol.

Imaging data analysis

ROI morphological analysis: to avoid methodological bias two different approaches were applied for the measurement of total surface areas in regions of interest (ROI: Follicular Areas). First, ROIs were analyzed with the Image J software. All images were transformed to binary, and two univariate morphological features were measured for each ROI: Surface area (Sa) and Circularity. Then, Surface area was transformed/normalized based on the scale bar of each binary image by using the formula Sa_{NORM} = Sa*Pixels of scale bar/Length of scale bar, in order to be able to compare ROIs obtained from different tissues. Next, trends were compared with those obtained from Imaris with the Surface Creation module, to ensure the two methods yielded comparable results before undertaking further within-ROI quantifications with Imaris. Furthermore, follicles

that had clear signs of damage (missing cellularity as determined by nuclear JOPRO-1 staining, tissue breaks, folds etc) were excluded from our analysis.

Histocytometry analysis: briefly, 3-dimensional segmented surfaces (based on nuclear signal) of background and spillover corrected images were generated with Imaris via the Surface Creation module. Average voxel intensities for all channels, as well as volume and sphericity of the 3-dimensional surfaces generated from histocytometry were exported in Microsoft Excel format. After conversion of the files to comma separated value (.CVS) files, the data were imported into FlowJo (version 10) for further analysis/quantification. Analysis included well-defined areas devoid of background staining or fragmentation and data were quantified either as relative frequencies or as cell counts normalized to total follicular area (mm^2) screened.

Neutralization Assays

The neutralization activity of plasma was measured by incubating 10µl of five-fold serially diluted serum in cDMEM with 40ul of diluted HIV-1 Env-pseudotyped virus for 30 minutes at 37ºC in a 96-well CulturPlate (Perkin Elmer). 20 µl of TZM-bl cells (10,000 cells/well) with or without 70µg/ml DEAE-Dextran was then added and incubated overnight at 37ºC. Each experiment plate also had a column of cells only (no Ab or virus) and a column of virus only (no Ab) as controls for background TZM-bl luciferase activity and maximal viral entry, respectively. Serial dilutions were performed with a change of tips at each dilution step to prevent carryover. The following day, all wells received 100µl of fresh cDMEM and were incubated overnight at 37ºC. The following day, 50µl of Steadylite Plus Reporter Gene Assay System (PerkinElmer) was added to all wells, and plates were shaken at 600RPM for 15 minutes. Luminometry was then performed on a SpectraMax L (Molecular Devices) luminometer. Percent neutralization is determined by calculating the difference in average RLU between virus only wells (cells + virus column) and test

wells (cells + plasma/Ab sample + virus), dividing this result by the average RLU of virus only wells (cell + virus column) and multiplying by 100. Background is subtracted from all test wells using the average RLU from the uninfected control wells (cells only column) before calculating the percent neutralization. Neutralizing plasma or serum antibody titers are expressed as the dilution required to achieve 50% neutralization (ID50) and calculated using a dose-response curve fit with a 5-parameter nonlinear function. For antibody class prediction, the neutralizing activity data were imported into the NFPws Neutralization Fingerprinting Web Server (http://iglab.accre.vanderbilt.edu/NFPws/) and analyzed as previously described [25].

Single-cell RNA-seq processing (scRNAseq) and donor-library integration

scRNAseq was performed on LN mononuclear cells from N (n = 4) and NNs (n = 4). The 3' V3 chemistry kit (10X Genomics) was used according to manufacturer's recommendations. Briefly, single cell Gel Beads-in-Emulsion (GEMs) were generated by combining 10,000 LN cells, barcoded Single Cell 3' v3.1 beads, and partitioning oil. This reaction was then run on a 10X Chromium Controller machine (10X Genomics). Afterwards, GEMs were processed to remove gel beads, and cells were lysed and underwent a reverse transcription reaction in a C1000 Touch Thermal Cycler (BioRad) to generate barcoded cDNA. The GEM mixture was then cleaned using Dynabeads to remove oil and cDNA was amplified. Amplified cDNA was then cleaned using the SPRIselect reagent and the quality/quantity of cDNA was assessed using a 2100 Bioanalyzer Instrument (Agilent Technologies). This cDNA then underwent fragmentation, end repair, A-tailing, and adaptor ligation according to manufacturer protocol recommendations. Library quality was assessed using Bioanalyzer. Libraries were then sent for sequencing at the Beijing Genomics Institute (BGI). Libraries were sequenced on a DNBseq-T7 (MGI Tech) machine targeting 20,000 reads per cell. To refine the annotation and improve cluster fidelity, we re-ran the SingleR

reference-based classification with increased stringency (quantile = 0.99, prune = TRUE, fine.tune = TRUE, tune.thresh = 0.99, de.method = “t”). Ambiguous T cells were reclassified based on expression thresholds for CD4 and CD8A/B genes (top 20th percentile). These refinements improved delineation between $CD4^+$ and $CD8^+$ subsets and resolved mixed annotations. Each donor sample was processed as an independent 10x Genomics scRNA-seq library. The applied pipeline for data analysis is the following: i) per-library QC and doublet removal (DoubletFinder) on each donor library, ii) SCTransform normalization with regression of percent.mt to remove the sample-level mitochondrial fraction, iii) PCA on the SCT residuals (top 30 PCs), iv) Harmony integration across donor libraries and v) Shared-nearest-neighbor graph and UMAP were then computed on the Harmony reduction. For UMAPs generation, Harmony was run on the SCTransform PCA across the *Donor* covariate and UMAPs were re-derived on the Harmony reduction. Cell-type annotations were generated by SingleR against the Monaco immune reference (main + fine labels) on the log-normalized RNA assay, followed by the refinement rules described. With respect to HIV mRNA detection, the 2024-A human genome assembly and transcript annotations were obtained from 10xgenomics.com. The HIV (HXB2 strain) reference genome (GenBank: K03455.1) was obtained from NCBI and appended as a chromosome to the human genome assembly and as a gene to the human GTF file. A custom reference was generated using Cell Ranger (v9.0.0) with the mkref command, followed by read counting using the count command. With respect to tonsillar transcriptomic analysis, we checked expression of key marker genes using the Human Tonsil Atlas (doi: 10.1016/j.immuni.2024.01.006) using the Seurat RNA-seq object downloaded from https://zenodo.org/records/8373756. Counts cells with labels matching “Tfh” or “T helper” were pseudobulked across patients and counts per million were calculated using edgeR version 4.6.3 (https://doi.org/10.1093/bioinformatics/btp616).

High-throughput, single-genome amplification and sequencing (HT-SGS) of HIV *env*

For reverse-transcription of the virus RNA, SuperScript III (ThermoFisher Scientific, 18080093) was used along with a gene specific reverse transcription primer, which is composed of an RNA binding site, 8 random-base Unique Molecular Identifiers (UMIs) and a reverse primer binding site for PCR (CCCGCGTGGCCTCCTGAATTATNNNNNNNNGTCATTGGTCTTAAAGGTACCTG). After RNA denaturation at 65 °C for 10 min, the RNA was reverse transcribed under following conditions: 50 °C for 10 min, 85 °C for 10 min, and 4°C hold. The cDNA was purified with a 2.2:1 volumetric ratio of RNAClean XP solid phase reverse immobilization beads (A63987, Beckman Coulter). The amount of cDNA was determined through limiting-dilution PCR using fluorescence-assisted clonal amplification (FCA)[89] to quantify cDNA copies. Next, the full-length *env* gene region was amplified in PCR for library preparation using the Advantage 2 PCR kit (Takara Bio, 639206) and forward (GAGCAGAAGACAGTGGCAATGA) and reverse (CCCGCGTGGCCTCCTGAATTAT) primers. The thermocycling conditions were as follows: initial denaturation at 95˚C for 1 min, 31 cycles of denaturation at 95˚C for 10 sec, annealing at 64˚C for 30 sec, and extension at 68˚C for 3 min, and final extension at 68˚C for 10 min. The final concentration of each reagent in PCR was 400 nM of forward and reverse primers, 200 mM of dNTP, 1X of Advantage 2 Buffer and 1X of Advantage 2 Polymerage Mix. Amplified products of approximately 3 kilobases were incorporated into sequencing libraries using the SMRTbell Express Template Prep Kit 2.0 (100-938-900, Pacific Biosciences) and Barcoded Overhang Adapter kit 8A and 8B (101-628-400 and 101-628-500, Pacific Biosciences) for multiplexing targeted sequencing. The libraries were treated by primer annealing and polymerase binding using the Sequel II Binding Kit 2.0 and Int Ctrl 1.0 (101-842-900, Pacific Biosciences), and then sequenced on a Sequel II system (Pacific Biosciences) with a 30-hour movie time under circular

consensus sequencing (CCS) mode. Bioinformatic analysis of sequence data was performed using a custom bioinformatic pipeline presented at https://github.com/niaid/UMI-pacbio-pipeline, with modifications to accommodate HIV *env* sequence length and primer sequences. Processed *env* sequences produced by this pipeline were translated and aligned with MAFFT [90] and manually curated. Pal2Nal was used to codon align the cDNA sequences to the protein alignment [91]. Shannon entropy was calculated per position for each position and also distinguishing synonymous and nonsynonymous positions (positions which could have both synonymous and nonsynonymous changes were not included in the synonymous/non-synonymous groups).

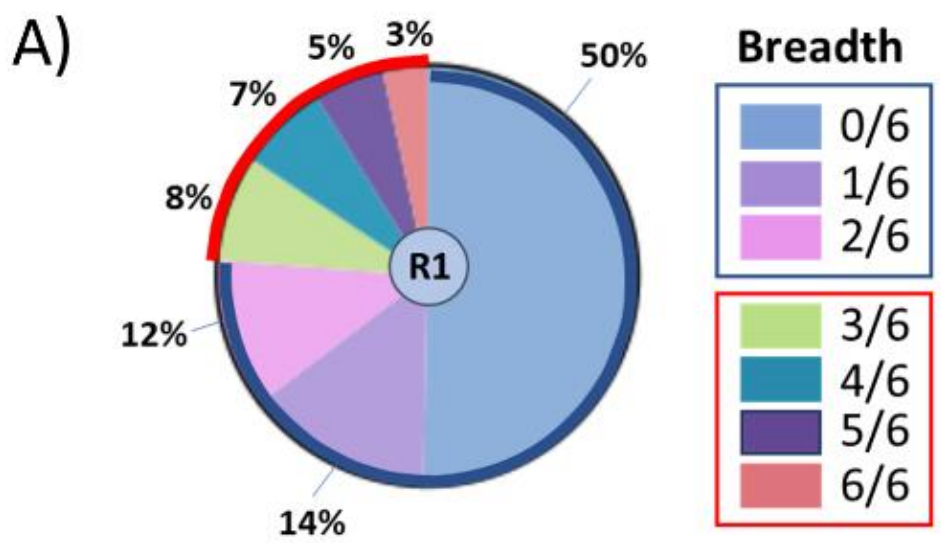

B) Cross-neutralization profiles of individual donors in the

	A	A	AD	AE	AE	AE	AE	AE	AG	B	B	BC	BC	C	C	C	C	C	D	D
PID	KER2008.12.SG3	Q259.17.SG3	Q461.e2.SG3	620345.c1.SG3	CNE5.SG3	CNE55.SG3	M02138.SG3	TH976.17.SG3	242-14.SG3	7165.18.SG3	YU2.DG.SG3	CH038.12.SG3	CH070.1.SG3	0013095-2.11.SG3	001428-2.42.SG3	26191-2.48.SG3	3168.V4.C10.SG3	ZM135.10a.SG3	231965.c1.SG3	247-23.SG3
Non neutralizers	(n=16)																			
HIV #1	<20	<20	<20	<20	<20	<20	<20	<20	<20	<20	<20	22	<20	<20	<20	<20	<20	<20	<20	<20
HIV #2	<20	<20	<20	<20	<20	<20	<20	<20	<20	<20	<20	<20	<20	<20	<20	<20	<20	<20	54	<20
HIV #3	<20	<20	<20	<20	<20	<20	<20	63	<20	28	<20	<20	<20	<20	<20	<20	<20	<20	<20	<20
HIV #4	<20	<20	<20	<20	<20	<20	<20	<20	<20	<20	<20	<20	<20	<20	<20	<20	<20	<20	<20	<20
HIV #5	<20	<20	<20	<20	<20	<20	<20	<20	<20	<20	<20	<20	<20	<20	<20	<20	<20	<20	<20	<20
HIV #6	<20	<20	<20	<20	<20	<20	<20	<20	22	64	<20	<20	<20	<20	<20	<20	<20	<20	<20	<20
HIV #7	<20	<20	<20	<20	<20	<20	<20	<20	35	54	<20	<20	<20	<20	<20	<20	<20	26	<20	<20
HIV #8	<20	<20	<20	<20	<20	<20	<20	<20	<20	<20	<20	<20	<20	<20	<20	<20	<20	26	<20	<20
HIV #9	<20	<20	<20	<20	<20	<20	<20	<20	<20	<20	<20	<20	<20	<20	<20	<20	<20	67	<20	<20
HIV #10	40	<20	<20	<20	<20	<20	<20	<20	<20	27	<20	<20	<20	<20	<20	<20	<20	117	<20	<20
HIV #11	<20	<20	<20	<20	<20	<20	<20	<20	26	22	<20	<20	26	<20	<20	<20	<20	23	<20	<20
HIV #12	<20	<20	<20	<20	<20	<20	<20	<20	<20	<20	<20	<20	<20	<20	<20	<20	<20	<20	<20	<20
HIV #13	<20	<20	<20	<20	<20	<20	<20	<20	21	<20	<20	<20	<20	<20	<20	<20	<20	<20	<20	<20
HIV #14	<20	<20	<20	<20	<20	<20	<20	<20	<20	<20	<20	<20	<20	<20	<20	<20	<20	<20	<20	<20
HIV #15	27	<20	<20	<20	<20	<20	<20	<20	<20	<20	<20	<20	<20	<20	<20	<20	<20	<20	<20	<20
HIV #16	<20	<20	<20	<20	<20	<20	<20	<20	<20	<20	<20	<20	<20	<20	<20	<20	<20	21	<20	<20
Neutralizers	(n=17)																			
HIV #17	39	42	<20	<20	32	<20	137	146	53	68	40	62	<20	185	83	24	<20	35	<20	<20
HIV #18	<20	<20	<20	<20	<20	<20	76	39	75	<20	<20	<20	<20	70	<20	<20	<20	53	<20	<20
HIV #19	<20	<20	<20	<20	<20	63	464	91	28	45	26	<20	<20	112	<20	21	<20	52	<20	<20
HIV #20	77	<20	<20	<20	<20	<20	232	180	322	38	73	<20	<20	90	44	<20	<20	54	<20	82
HIV #21	<20	<20	<20	<20	<20	<20	115	118	51	102	<20	32	<20	240	<20	<20	<20	82	<20	23
HIV #22	32	86	<20	<20	<20	<20	<20	27	<20	<20	33	96	150	27	41	60	<20	<20	<20	78
HIV #23	345	76	51	82	58	176	130	54	129	262	129	255	360	179	170	43	54	153	631	<20
HIV #24	83	<20	<20	111	<20	175	381	169	137	300	213	102	<20	160	35	38	<20	328	28	<20
HIV #25	76	64	<20	52	<20	56	105	64	83	70	235	<20	<20	139	895	28	154	183	20	<20
HIV #26	<20	161	<20	36	27	95	150	97	57	91	135	<20	39	177	85	32	<20	283	35	<20
HIV #27	782	198	<20	78	109	107	1,046	506	1,283	1,147	195	94	154	142	537	34	100	230	430	208
HIV #28	<20	174	<20	<20	<20	<20	42	154	97	114	<20	<20	76	<20	<20	<20	25	149	<20	<20
HIV #29	<20	<20	128	<20	<20	<20	<20	54	117	146	<20	<20	<20	288	<20	<20	<20	280	<20	<20
HIV #30	<20	<20	<20	<20	<20	31	<20	<20	<20	266	389	496	231	<20	<20	<20	<20	<20	<20	<20
HIV #31	<20	<20	<20	381	134	479	545	177	511	37	31	<20	<20	54	85	<20	<20	<20	662	27
HIV #32	42	<20	<20	<20	<20	83	<20	<20	71	96	145	<20	<20	177	22	<20	<20	<20	33	<20
HIV #33	23	<20	<20	<20	<20	<20	<20	<20	72	100	137	47	<20	311	<20	<20	<20	74	<20	<20

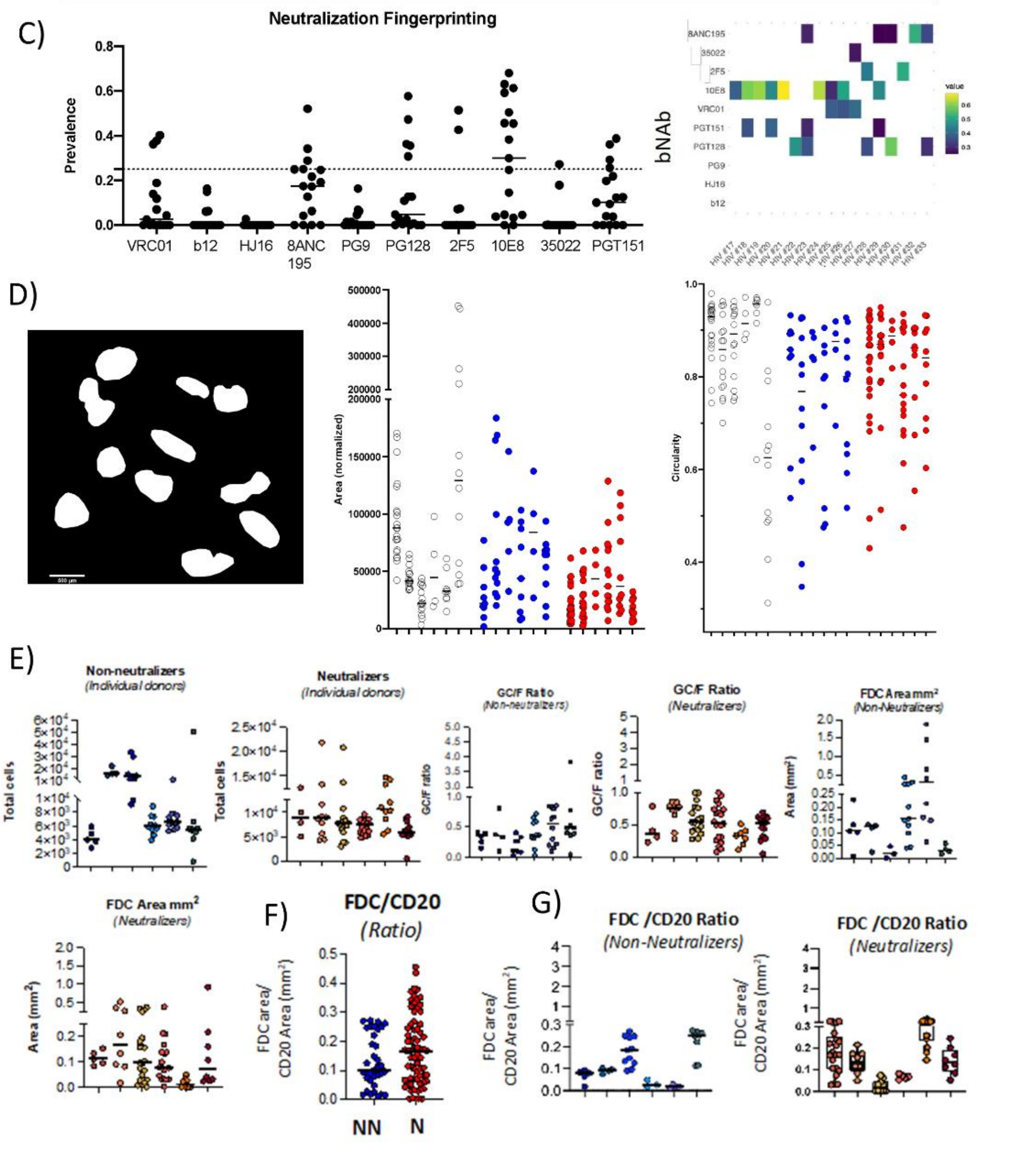

Supplemental Figure 1: **Cross-neutralization profiles of NN and N study donors, and heterogeneity of B-cell follicle shape**. A) The distribution of cross-neutralization breadth in the population of study defined as the percentage of the number of 6 isolates (mini panel) recognized among study participants B) Profiles of plasma neutralizing activity (ID50) in the 33 HIV + donors included in the study color coded by magnitude (green=low, yellow=medium, orange=high). Plasmas were assayed against a panel of 20 different HIV isolates. C) Dot plot showing the distribution of predicted bNAb lineages in the serum of neutralizers as calculated with the NFPws Neutralization Fingerprinting Web Server (http://iglab.accre.vanderbilt.edu/NFPws/) and Heatmap showing the number of bNAbs with a prevalence coefficient value >0.25 in each donor with neutralizing activity. Note that for visualization purposes only those values that were found to be > 0.25 are shown. D) Representative example of a binary image used for area determination with ImageJ/Fiji; the areas and circularity in Healthy controls (white circles), NNs (blue circles) and Ns (red circles) are shown. Each circle represents an individual follicle in the tissue under study. E-G) Dot plots showing the heterogeneity of Total cells, GC/F ratios and FDC areas (mm^2) in each donor under study, as well as FDC/CD20 ratios in NNs (blue circles, n=6) and Ns (red circles, n=6) as calculated using data from pooled quantitative imaging analysis and Histocytometry, as well as the heterogeneity of FDC/CD20 ratios in NNs and Ns as measured by Histocytometry (p=0.144). Circles represent individual follicles. p values represent probabilities as calculated by an ANOVA. Median values are shown in the dot plots.

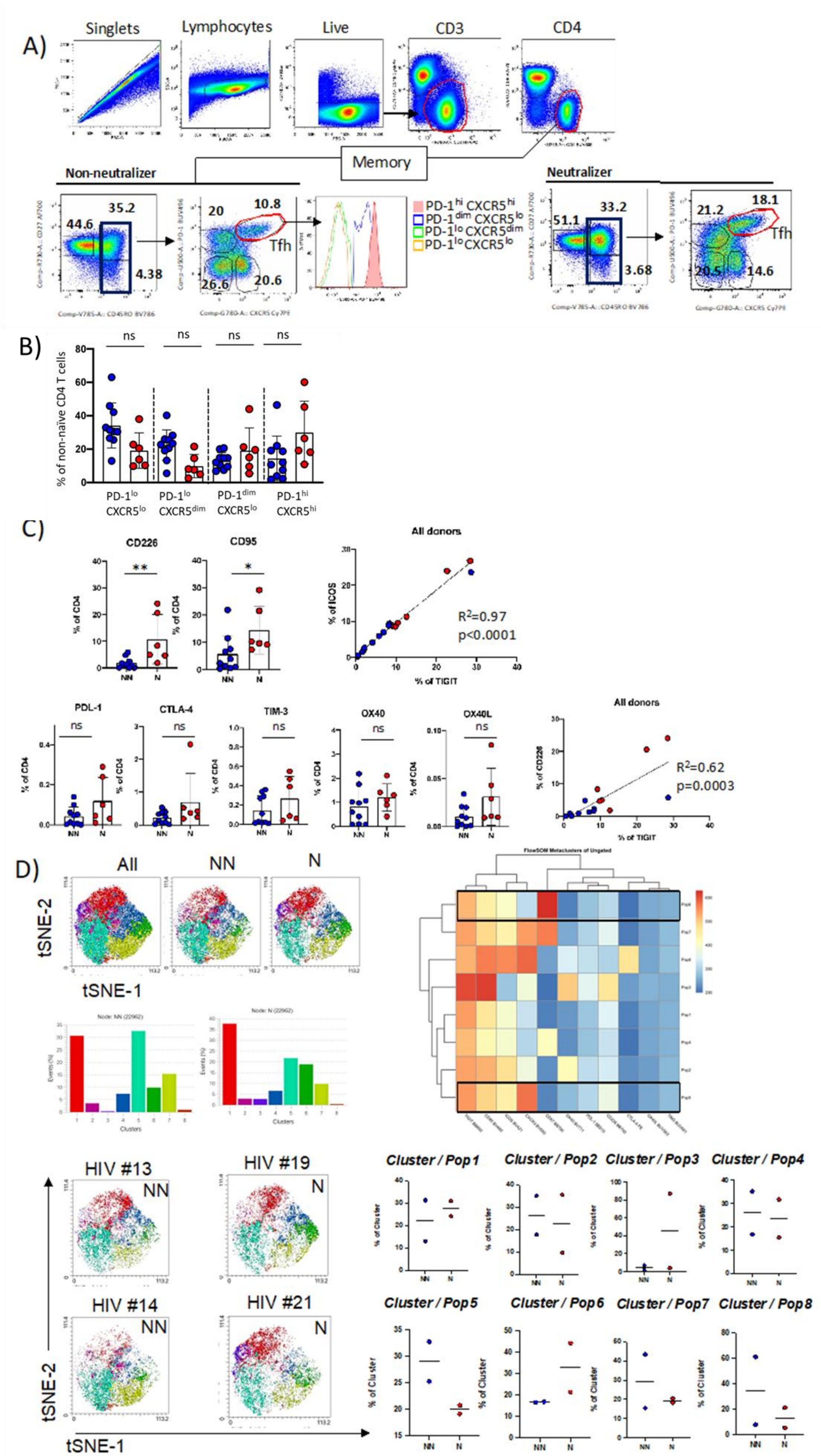

A)
Singlets
Lymphocytes
Live
CD3
CD4
Memory
Non-neutralizer
Neutralizer
44.6
35.2
4.38
20
10.8
26.6
20.6
Tfh
PD-1hi CXCR5hi
PD-1dim CXCR5lo
PD-1lo CXCR5dim
PD-1lo CXCR5lo
51.1
33.2
3.68
21.2
18.1
20.5
14.6
B)
% of non-naive CD4 T cells
ns
PD-1lo CXCR5lo
PD-1lo CXCR5dim
PD-1dim CXCR5lo
PD-1hi CXCR5hi
C)
CD226
CD95
% of CD4
NN
N
All donors
% of ICOS
% of TIGIT
R2=0.97
p<0.0001
PDL-1
CTLA-4
TIM-3
OX40
OX40L
% of CD226
R2=0.62
p=0.0003
D)
All
NN
N
tSNE-2
tSNE-1
FlowSOM Metaclusters of Ungated
Node: NN (22902)
Node: N (22902)
Events (%)
Clusters
HIV #13
HIV #19
HIV #14
HIV #21
Cluster / Pop1
Cluster / Pop2
Cluster / Pop3
Cluster / Pop4
Cluster / Pop5
Cluster / Pop6
Cluster / Pop7
Cluster / Pop8
% of Cluster

Supplemental Figure 2: **Phenotypic characterization of T_{FH} cells**. A) Gating strategy for T_{FH} cell characterization in LN cell suspensions using flow cytometry. B) Cumulative flow cytometry frequencies of PD-1$^{lo/dim/hi}$ CXCR5 $^{lo/dim/hi}$ populations within the CD27$^{hi/lo}$ CD45RO^{hi} CD4 T cell memory compartment in NNs and Ns. C) Cumulative flow cytometry frequencies of inhibitory and activating receptors CD226, CD95, PDL-1, CTLA-4, TIM-3, OX40, and OX40L within the CD4 T cell compartment in NNs and Ns, and association of TIGIT expression with ICOS and CD226. CD226 **p= 0.0075. CD95 *p=0.0312 (Mann Whitney U test). Mean ±SD values are shown. D) Overlays showing the 8 T_{FH} cell clusters identified through t-SNE (t-stochastic neighbor embedding) analysis of flow cytometry using data from 4 samples with sufficiently representative TFH populations (NN: HIV#13, 14 and N: HIV#19, 21), and their relative representation in the 4 donors analyzed. Clustering was based on the markers TIGIT, ICOS, CD95, CD57, CXCR3, OX40, CTLA-4, PDL-1, CD226, OX40L and TIM-3.

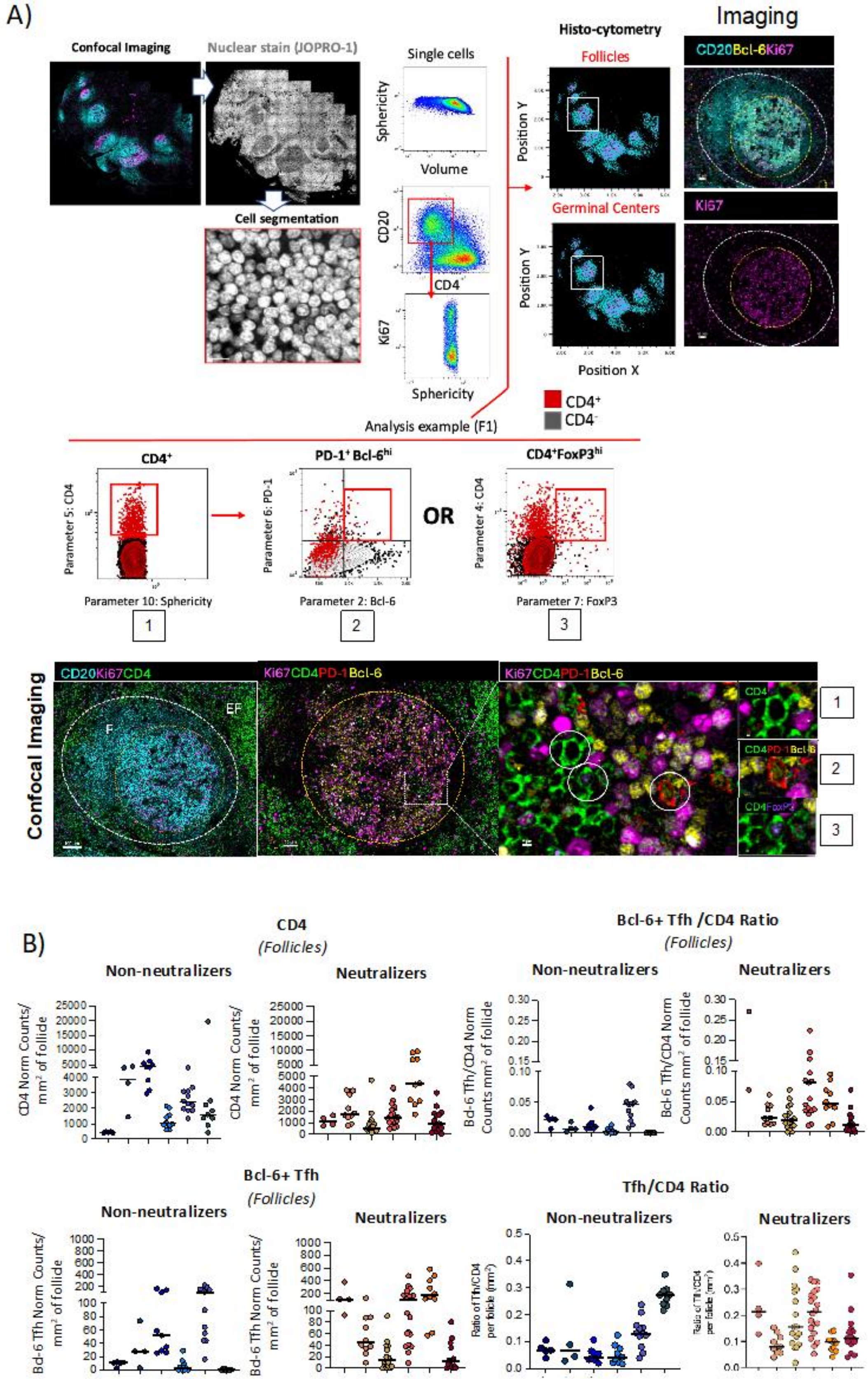

A)
Confocal Imaging
Nuclear stain (JOPRO-1)
Cell segmentation
Single cells
Sphericity
Volume
CD20
CD4
Ki67
Sphericity
Histo-cytometry
Follicles
Germinal Centers
Position Y
Position X
Imaging
CD20Bcl-6Ki67
Ki67
CD4+
CD4-
Analysis example (F1)
CD4+
PD-1+ Bcl-6hi
CD4+FoxP3hi
Parameter 5: CD4
Parameter 10: Sphericity
Parameter 6: PD-1
Parameter 2: Bcl-6
OR
Parameter 4: CD4
Parameter 7: FoxP3
1
2
3
Confocal Imaging
CD20Ki67CD4
Ki67CD4PD-1Bcl-6
Ki67CD4PD-1Bcl-6
F
EF
CD4
CD4PD-1Bcl-6
CD4FoxP3
B)
CD4
(Follicles)
Non-neutralizers
Neutralizers
CD4 Norm Counts/ mm2 of follicle
Bcl-6+ Tfh /CD4 Ratio
(Follicles)
Bcl-6 Tfh/CD4 Norm Counts mm2 of follicle
Bcl-6+ Tfh
(Follicles)
Bcl-6 Tfh Norm Counts/ mm2 of follicle
Tfh/CD4 Ratio
Ratio of Tfh/CD4 per follicle (mm2)

Supplemental Figure 3: ***In situ* characterization of T_{FH} cells**. A) Gating strategy used for subset characterization using Histocytometry. For the analysis, tissues were stained with anti-CD20 and anti-Ki67 for B cell follicle characterization as well as anti-PD1 and anti-Bcl-6 or FoxP3 in combination with CD4 for T_{FH} cells and T_{FR}/T_{REG} T cells respectively. The nuclear marker JOPRO-1 was also included to aid with computational segmentation. Confocal images were transformed to FlowJo files as previously described, and CD4, PD-1^{hi}Bcl-6^{hi} CD4 (Bcl-6 T_{FH}) and FoxP3^{hi} CD4 T cell frequencies within individual B cell follicles were calculated using FlowJo as shown. Original magnification at 40x (NA 1.3). B) Pooled data of normalized CD4 and Bcl-6 T_{FH} cell numbers within the B cell follicles of 6 NN and 6 N LNs. The ratios of Bcl-6 T_{FH} /CD4 and T_{FH}/CD4 for NNs and Ns is also shown. Each circle represents a single follicle, and each box plot represents tissue from a single donor. Normalization was performed by calculating the number of cells per mm^2 of area screened. Median values are shown in dot plots.

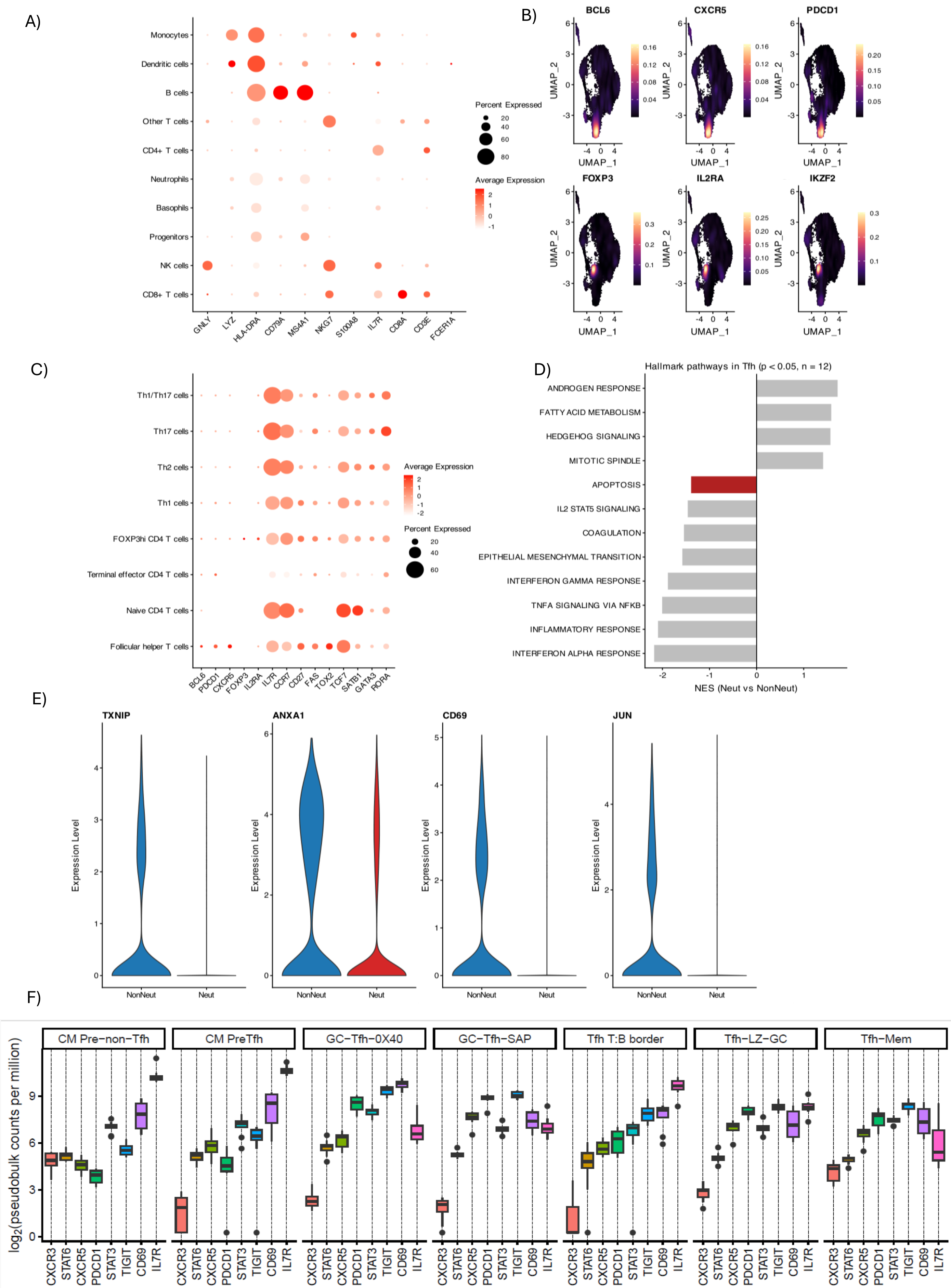

A)
Monocytes
Dendritic cells
B cells
Other T cells
CD4+ T cells
Neutrophils
Basophils
Progenitors
NK cells
CD8+ T cells
GNLY
LYZ
HLA-DRA
CD79A
MS4A1
NKG7
S100A8
IL7R
CD8A
CD3E
FCER1A
Percent Expressed
20
40
60
80
Average Expression
2
1
0
-1
B)
BCL6
CXCR5
PDCD1
FOXP3
IL2RA
IKZF2
UMAP_2
UMAP_1
C)
Th1/Th17 cells
Th17 cells
Th2 cells
Th1 cells
FOXP3hi CD4 T cells
Terminal effector CD4 T cells
Naive CD4 T cells
Follicular helper T cells
BCL6
PDCD1
CXCR5
FOXP3
IL2RA
IL7R
CCR7
CD27
FAS
TOX2
TCF7
SATB1
GATA3
RORA
Average Expression
Percent Expressed
D)
Hallmark pathways in Tfh (p < 0.05, n = 12)
ANDROGEN RESPONSE
FATTY ACID METABOLISM
HEDGEHOG SIGNALING
MITOTIC SPINDLE
APOPTOSIS
IL2 STAT5 SIGNALING
COAGULATION
EPITHELIAL MESENCHYMAL TRANSITION
INTERFERON GAMMA RESPONSE
TNFA SIGNALING VIA NFKB
INFLAMMATORY RESPONSE
INTERFERON ALPHA RESPONSE
NES (Neut vs NonNeut)
E)
TXNIP
ANXA1
CD69
JUN
Expression Level
NonNeut
Neut
F)
CM Pre-non-Tfh
CM PreTfh
GC-Tfh-0X40
GC-Tfh-SAP
Tfh T:B border
Tfh-LZ-GC
Tfh-Mem
log2(pseudobulk counts per million)
CXCR3
STAT6
CXCR5
PDCD1
STAT3
TIGIT
CD69
IL7R

Supplementary Figure 4. Lymph node and CD4 T cell subset cell-type marker-gene annotations and Pathway analysis in neutralizer vs non-neutralizer TFH cells. A) DotPlot of canonical lineage marker genes (*CD79A*, *MS4A1*, *CD3E*, *IL7R*, *CD8A*, *HLA-DRA*, *S100A8*, *LYZ*, *FCER1A*, *GNLY*, *NKG7*) across the main cell-type labels. Cell-type rows and gene columns were hierarchically clustered on the average expression matrix. Dot size encodes the percentage of cells in the cluster expressing the gene; color encodes the z-scored average expression across clusters. B) Weighted kernel-density-estimate (KDE) plots of representative CD4 T-cell subset markers on the post-Harmony CD4 T-cell UMAP: BCL6, CXCR5 and PDCD1 for TFH cells; FOXP3, IL2RA and IKZF2 for FOXP3^{hi} CD4 T cells. For each gene, cells were resampled with probability proportional to log-normalized expression and a 2D KDE was computed then interpolated back to each cell's UMAP coordinates. The color scale encodes local expression-weighted density. C) DotPlot of CD4 T-cell subset-defining markers (*BCL6*, *PDCD1*, *CXCR5*, *FOXP3*, *IL2RA*, *IL7R*, *CCR7*, *CD27*, *FAS*, *TOX2*, *TCF7*, *SATB1*, *GATA3*, *RORA*) across CD4 fine annotations (TFH cells, Naive CD4 T cells, Terminal effector CD4 T cells, FOXP3^{hi} CD4 T cells, Th1 cells, Th2 cells, Th17 cells and Th1/Th17 cells). Cell-type rows are hierarchically clustered. Dot size and color scale as in (A). D) Hallmark gene-set enrichment in TFH cells, neutralizers vs non-neutralizers. Bar plot of normalized enrichment scores (NES) for all Hallmark pathways with nominal $p < 0.05$ in the TFH contrast (fgsea, gene rank = signed –log10 p from MAST DE, *Neut* vs *NonNeut*). Positive NES = up-regulated in neutralizers; negative NES = up-regulated in non-neutralizers. HALLMARK_APOPTOSIS is highlighted in red. E) Violin plots of the four representative leading-edge apoptosis genes, TXNIP, ANXA1, CD69 and JUN in TFH cells, split by Group (NonNeut, Neut). Each violin shows the distribution of log-normalized expression across all TFH cells from the corresponding Group; y-axis: *Expression Level* (Seurat default log-normalized

counts). F) Transcriptomic characterization of tonsillar CD4 T cell subsets. An online available data set using tonsillar cells (HCATonsilData) was used for the analysis of the expression of eight relevant genes. The log2 (pseudobulk) counts per million, with library size calculated across all T helper cells using edgeR, are shown.

A)

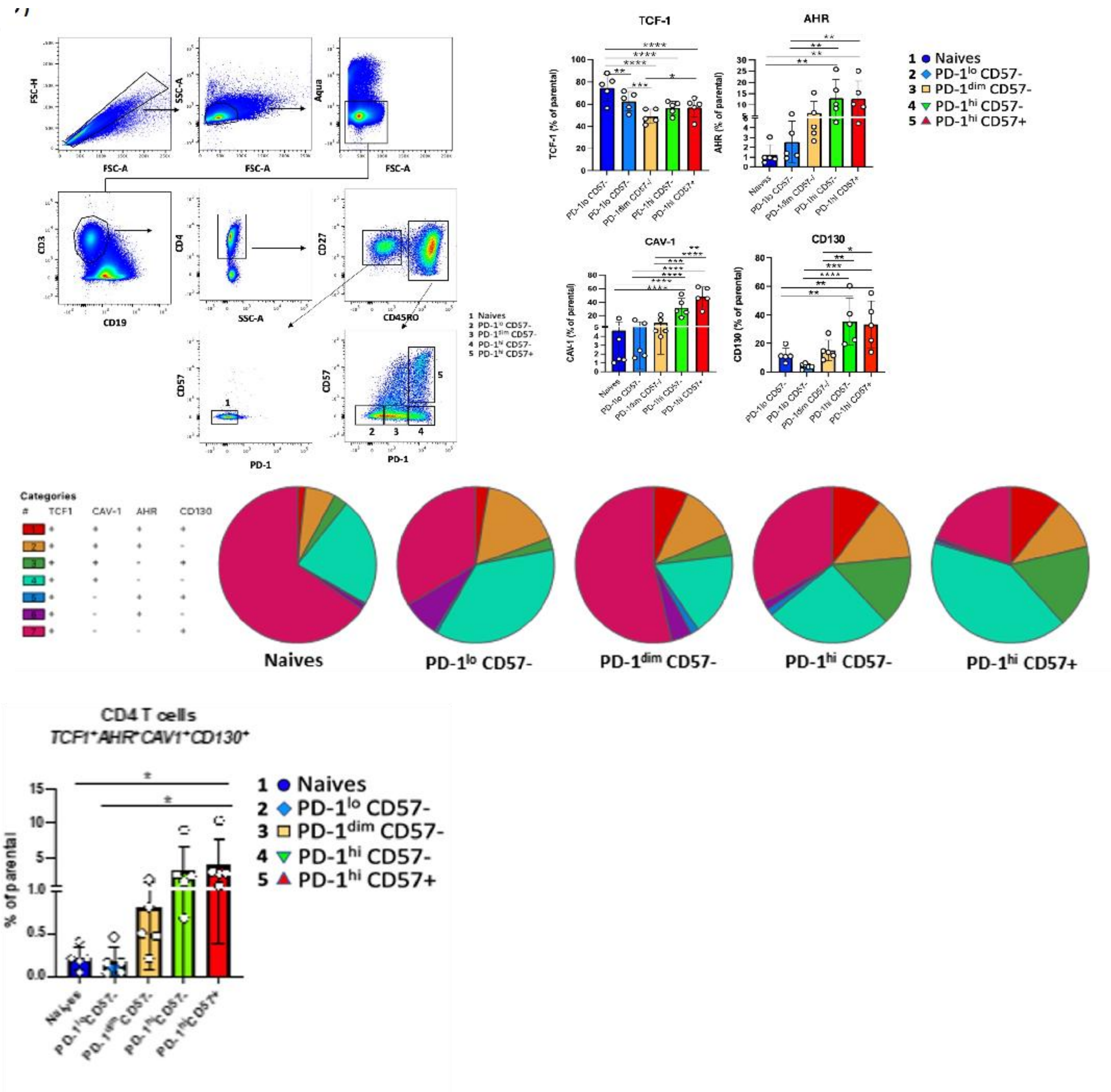

FSC-H
FSC-A
SSC-A
Aqua
CD3
CD19
CD4
CD27
CD45RO
CD57
PD-1
1 Naives
2 PD-1lo CD57-
3 PD-1dim CD57-
4 PD-1hi CD57-
5 PD-1hi CD57+
TCF-1
AHR
CAV-1
CD130
TCF-1 (% of parental)
AHR (% of parental)
CAV-1 (% of parental)
CD130 (% of parental)
Categories
TCF1 CAV-1 AHR CD130
Naives
PD-1lo CD57-
PD-1dim CD57-
PD-1hi CD57-
PD-1hi CD57+
CD4 T cells
TCF1+AHR+CAV1+CD130+
% of parental

B)

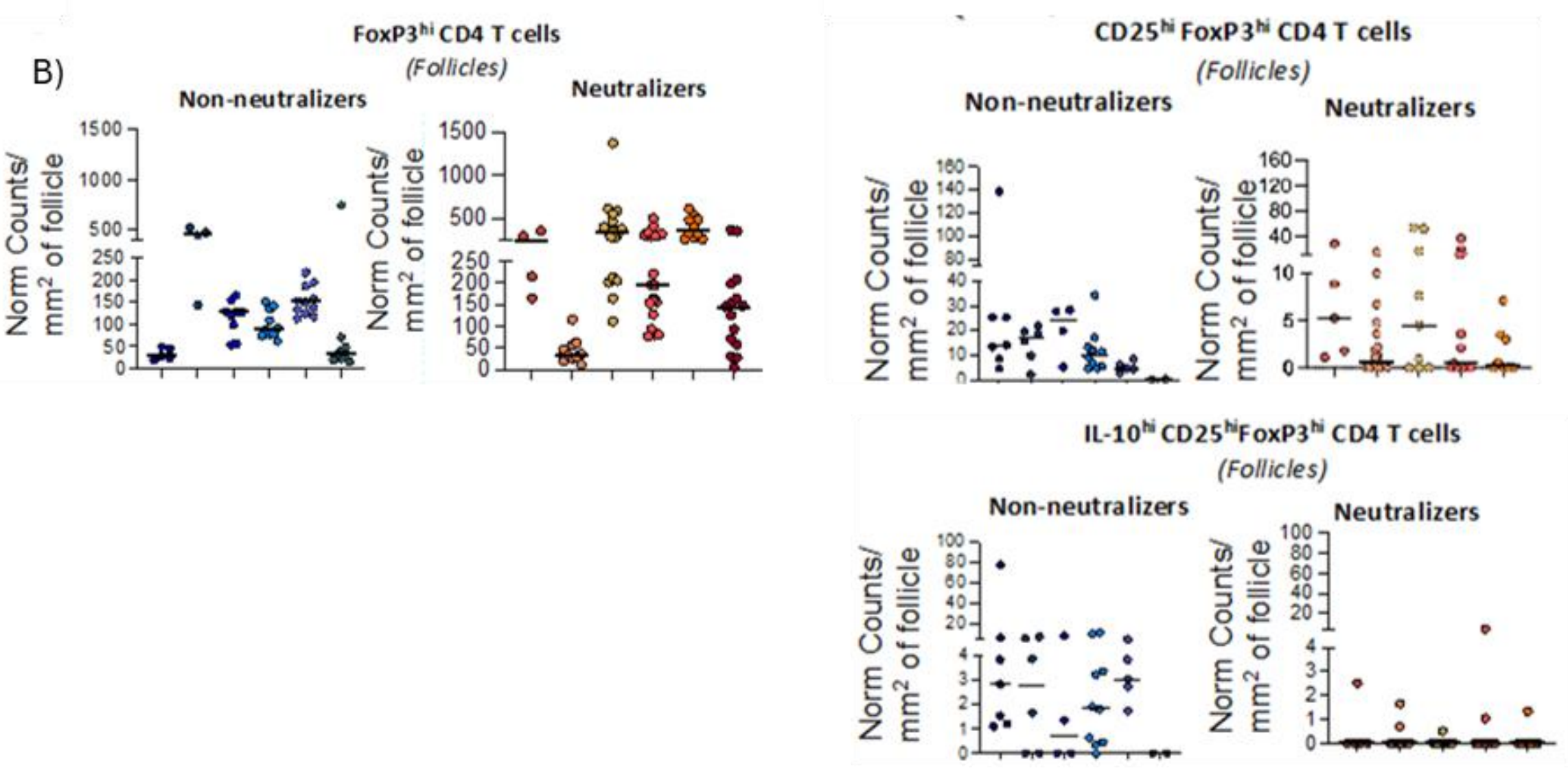

FoxP3hi CD4 T cells
(Follicles)
Non-neutralizers
Neutralizers
Norm Counts/ mm2 of follicle
CD25hi FoxP3hi CD4 T cells
(Follicles)
IL-10hi CD25hiFoxP3hi CD4 T cells
(Follicles)

Supplemental Figure 5: **Characterization of tonsillar single cell suspension with flow cytometry.** A) Flow cytometry gating strategy used for the characterization of CD57$^{lo/hi}$ T_{FH} cells and bar charts showing the frequencies of TCF-1, AHR, CAV-1 and CD130 expressing cells within each T_{FH} cell subpopulation as well as combined expression of these four markers and their relative proportion of co-expressing cells (pie charts). Data shown are pooled data from 5 tonsils and each circle represents a different tonsil. B) Cumulative normalized numbers of FoxP3^{hi} CD4 T cells, CD25^{hi} FoxP3^{hi} CD4 T cells and IL-10^{hi} CD25^{hi} FoxP3^{hi} CD4 T cells in NNs and Ns as measured by confocal imaging analysis and HistoCytometry. Each circle represents a B cell follicle and follicles from the same donor tissue are grouped together under the same color, with each box plot denoting a different donor. Median values are shown in dot plots.

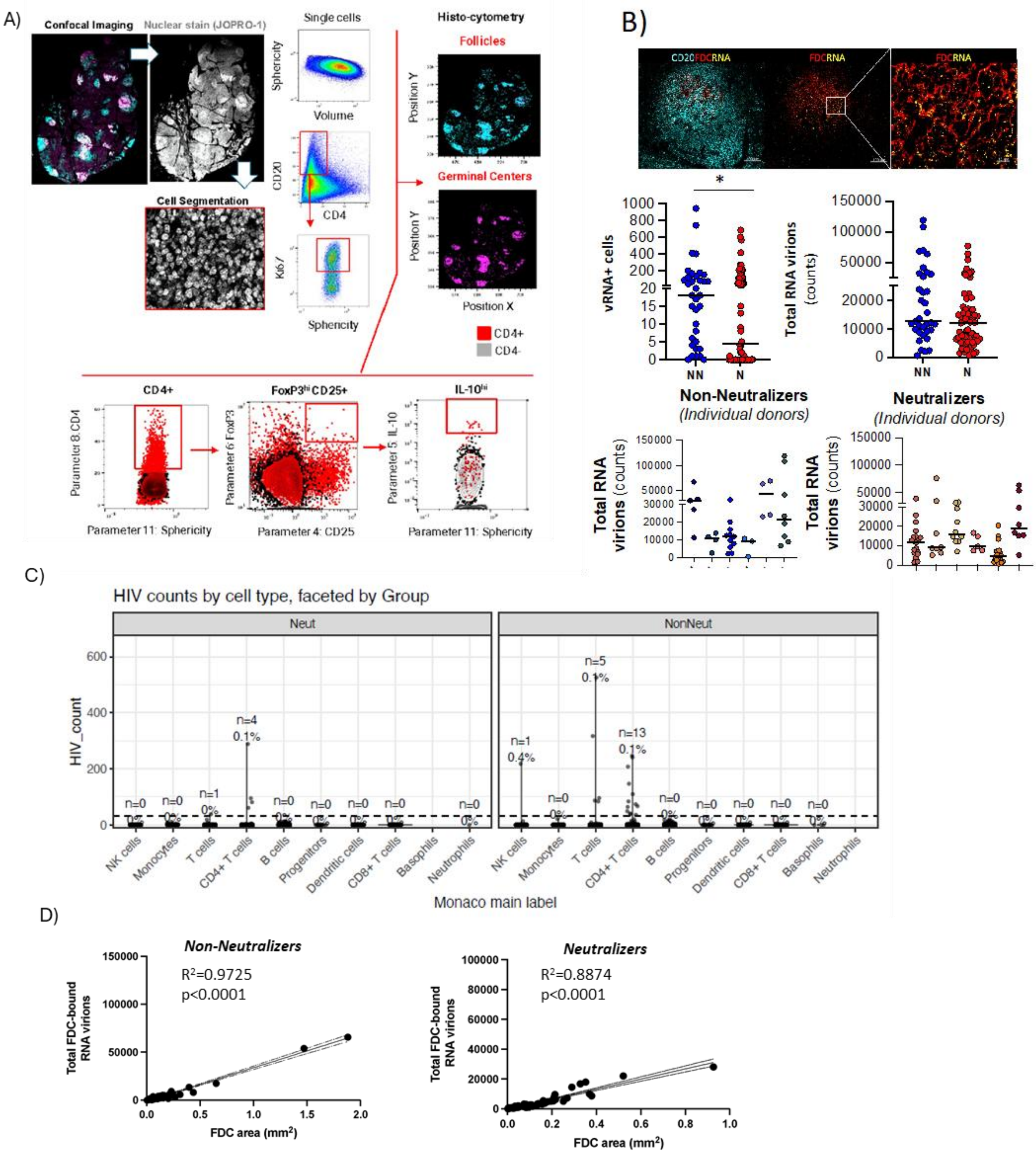
A)
Confocal Imaging
Nuclear stain (JOPRO-1)
Cell Segmentation
Single cells
Sphericity
Volume
CD20
CD4
Ki67
Histo-cytometry
Follicles
Germinal Centers
Position Y
Position X
CD4+
CD4-
FoxP3hi CD25+
IL-10hi
Parameter 8: CD4
Parameter 11: Sphericity
Parameter 6 FoxP3
Parameter 4: CD25
Parameter 5: IL-10
B)
CD20 FDC RNA
FDC RNA
vRNA+ cells
Total RNA virions (counts)
NN
N
Non-Neutralizers
(Individual donors)
Neutralizers
(Individual donors)
C)
HIV counts by cell type, faceted by Group
Neut
NonNeut
HIV_count
NK cells
Monocytes
T cells
CD4+ T cells
B cells
Progenitors
Dendritic cells
CD8+ T cells
Basophils
Neutrophils
Monaco main label
D)
Non-Neutralizers
R²=0.9725
p<0.0001
Neutralizers
R²=0.8874
p<0.0001
Total FDC-bound RNA virions
FDC area (mm²)

Supplemental Figure 6: In situ identification of regulatory T cells and virological measurements. A) Gating strategy used for regulatory T cell characterization by Histocytometry. For the analysis, tissues were stained with anti-CD20 (cyan) and anti-Ki67 (magenta) for B cell follicle characterization as well as anti-CD4, anti-CD25 and anti-FoxP3 in combination with IL-10 for the characterization of corresponding T_{Fr}/T_{REG} T cells. The nuclear marker JOPRO-1 was also included to aid with computational segmentation. Confocal images were transformed to FlowJo files as previously described, and $CD25^{hi}FoxP3^{hi}$ $IL\text{-}10^{hi}$ CD4 T cell frequencies (T_{FR}) within individual B cell follicles were calculated using FlowJo as shown. Original magnification at 40x (NA 1.3). B) Representative confocal images showing the distribution of viral RNA (yellow) on FDC networks (red) within B-cell follicles (CD20: cyan) and dot plots summarizing the number of RNA+ cells and vRNA+ virions as measured by RNAscope *in situ* hybridization, Histocytometry (cells) and spot analysis (virions) in NNs (n=6, blue circles represent all follicles counted in those six participants) vs Ns (n=6, red circles represent all follicles counted as for NNs). Images were captured at 40x magnification, with 1% zoom and scale is 100um and 15um (zoomed in detail). Each blue and red circle represents a single follicle, and data presented are for all follicles measured in NNs (blue) and Ns (red) tissues *p=0.0232 (Mann-Whitney U test); **p=0.0704 (ANOVA). Graphs showing the number of total RNA virions (counts) in individual NN and N participants are also shown. Each color represents a different participant tissue and individual circles the B-cell follicles in that tissue. C) Detection of HIV RNA–positive cells in lymph node scRNA-seq data. Single-cell RNA sequencing data from lymph nodes of neutralizers (Neut) and non-neutralizers (NonNeut) were re-aligned to the HIV genome to identify cells containing viral reads. Each bar represents the number and frequency of cells with detectable HIV reads across

major immune cell types. D) Association of total RNA virion burden on FDCs and total FDC area (mm^2) as measured by surface analysis. Median values are shown in dot plots.

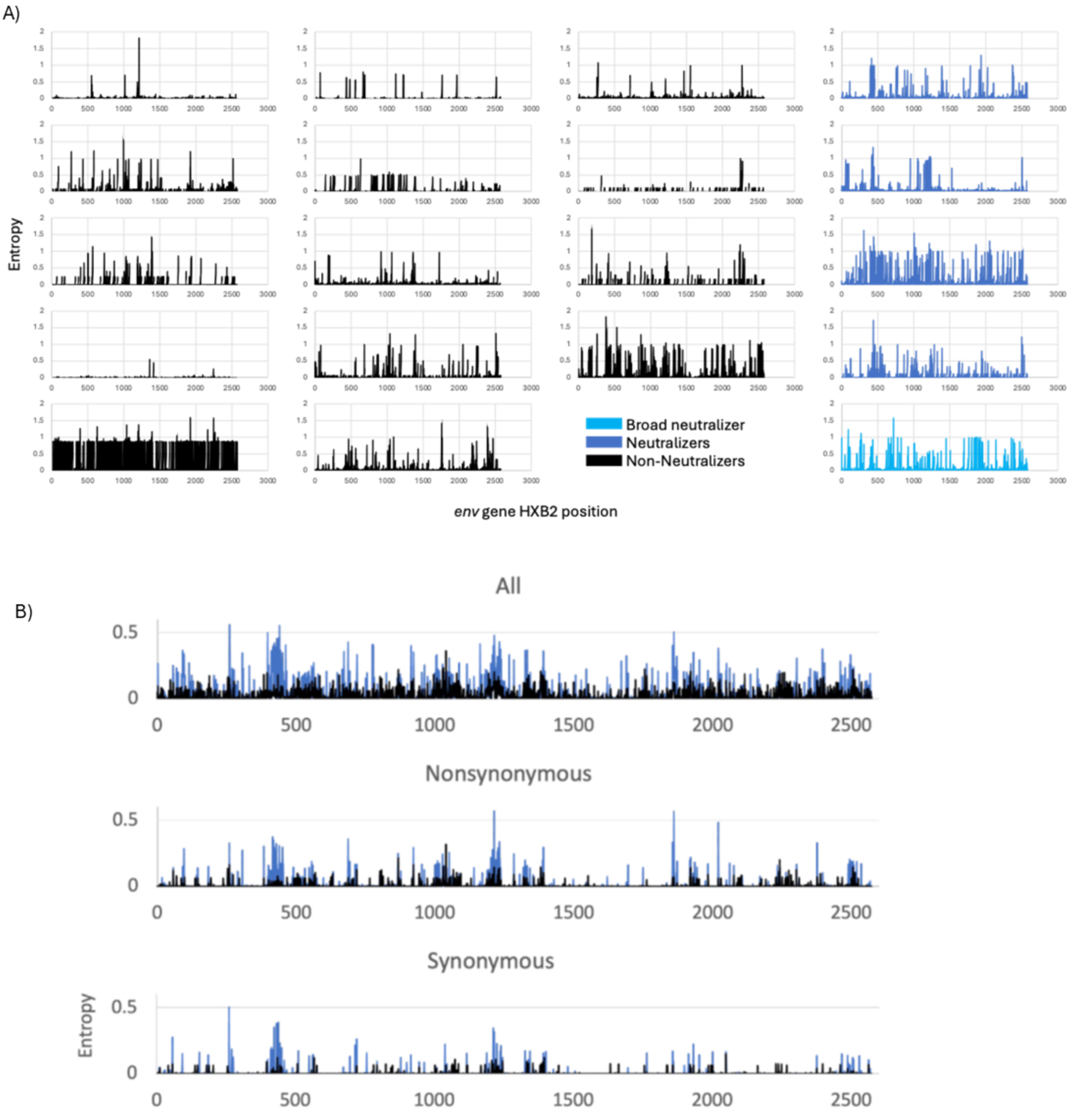

A)
Entropy
Broad neutralizer
Neutralizers
Non-Neutralizers
env gene HXB2 position
B)
All
Nonsynonymous
Synonymous
Entropy

Supplemental Figure 7: Evolution of HIV Env. A) Entropy-H(x) plot showing the location of positions with residue mismatches on alignment to the reference Env HIV sequence. The plots for each non-neutralizer, neutralizer and a broad neutralizer are shown. B) Entropy-H(x) plot showing the location of positions with residue mismatches for non-synonymous and synonymous residues in NNs (black) and Ns (cyan).

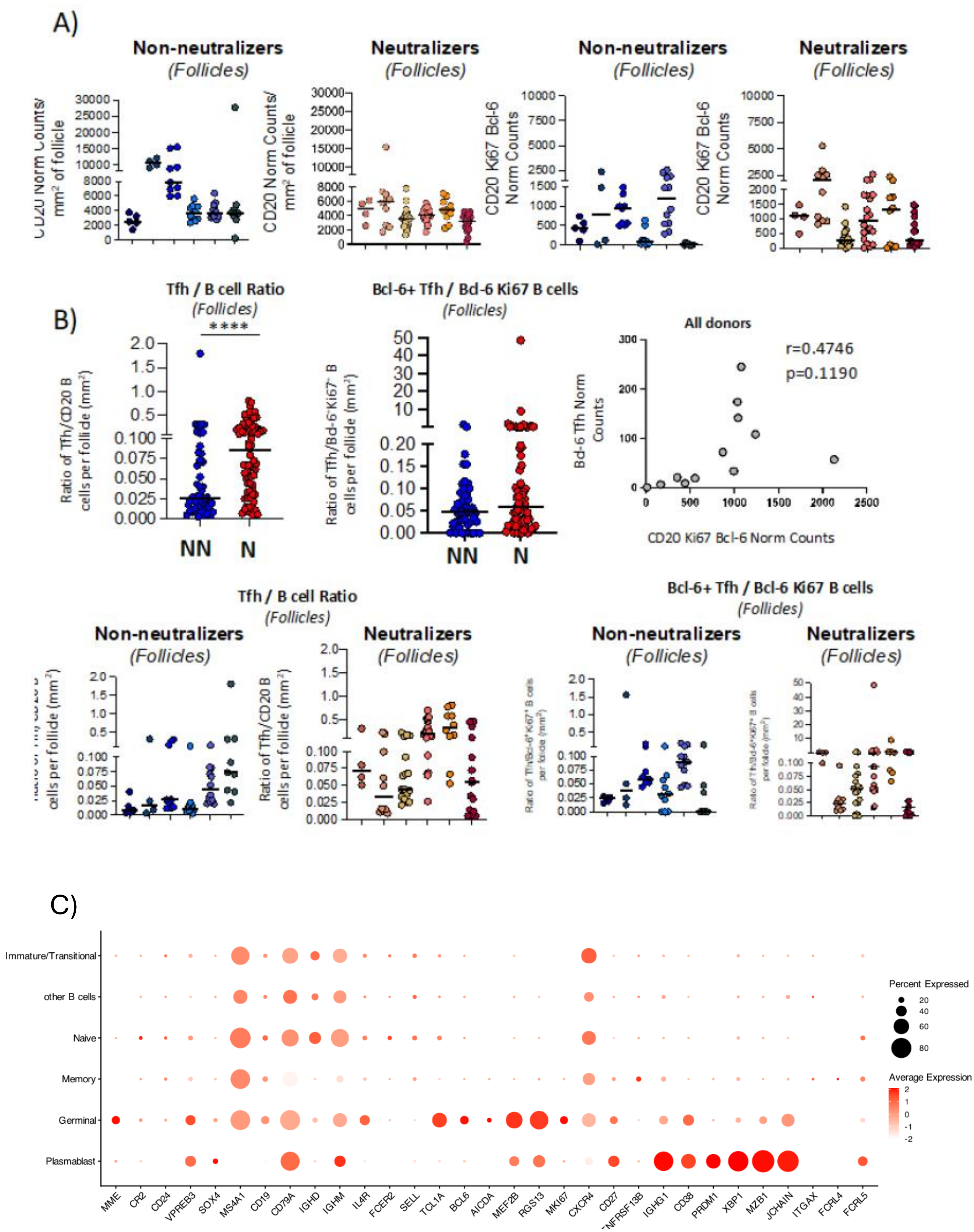
A)
Non-neutralizers
(Follicles)
Neutralizers
(Follicles)
Non-neutralizers
(Follicles)
Neutralizers
(Follicles)
CD20 Norm Counts/ mm² of follicle
CD20 Ki67 Bcl-6 Norm Counts
B)
Tfh / B cell Ratio
(Follicles)
****
Ratio of Tfh/CD20 B cells per follicle (mm²)
NN
N
Bcl-6+ Tfh / Bcl-6 Ki67 B cells
(Follicles)
Ratio of Tfh/Bcl-6⁺Ki67⁺ B cells per follicle (mm²)
All donors
r=0.4746
p=0.1190
Bcl-6 Tfh Norm Counts
CD20 Ki67 Bcl-6 Norm Counts
Tfh / B cell Ratio
(Follicles)
Bcl-6+ Tfh / Bcl-6 Ki67 B cells
(Follicles)
C)
Immature/Transitional
other B cells
Naive
Memory
Germinal
Plasmablast
MME
CR2
CD24
VPREB3
SOX4
MS4A1
CD19
CD79A
IGHD
IGHM
IL4R
FCER2
SELL
TCL1A
BCL6
AICDA
MEF2B
RGS13
MKI67
CXCR4
CD27
TNFRSF13B
IGHG1
CD38
PRDM1
XBP1
MZB1
JCHAIN
ITGAX
FCRL4
FCRL5
Percent Expressed
20
40
60
80
Average Expression
2
1
0
-1
-2

Supplemental Figure 8: B cell histocytometry analysis per donor and B cell subsets marker-gene annotations**.** A) Dot plots showing the normalized numbers of $CD20^{hi}$ and $CD20^{hi}Ki67^{hi}Bcl\text{-}6^{hi}$ B cells in NNs and Ns as measured by Histocytometry. Each donor is represented by a different color. Circles represent single follicles and follicles from the same tissue share the same color. B) Pooled analysis of B cell follicle T_{FH} cell/CD20 B cell ratios (left: ****$p<0.0001$) and T_{FH} cells / $Bcl\text{-}6^{hi}Ki67^{hi}$ B cells ($p=0.0568$) in NNs (blue circles, n=6) and Ns (red circles, n=6) as measured by confocal imaging and Histocytometry. Each circle represents a single B cell follicle and follicles from the same group are shown together (NNs: blue, Ns: red). Donor-specific data in NNs and Ns are also shown. Each donor is represented by a different color. Circles represent single follicles and follicles from the same tissue share the same color. The correlation between $Bcl\text{-}6^{hi}$ T_{FH} and $Bcl\text{-}6^{hi}Ki67^{hi}CD20^{hi}$ B cells numbers is shown too. Group differences between NNs and Ns were determined by One-Way ANOVA. Probability values $p<0.05$ were considered significant. Median values are shown in dot plots. C) DotPlot of canonical B-cell marker genes across the B-cell subsets used in Fig. 5B (Immature/Transitional, other B cells, Naive, Memory, Germinal, Plasmablast). Markers are arranged in B-cell developmental order, grouping transitional/immature markers, pan-B/naive markers, germinal-center markers, memory markers, plasmablast / plasma-cell markers, and atypical / activated B-cell markers. Dot size encodes Percent Expressed; color encodes the z-scored average expression across subsets.

Supplemental Table 1: Clinical Information of study participants

ID	Age	Gender	VL_{log}	%CD4	[CD4 count]	Treatment	Tissue Type	Histopathology
**Non neutralizers**								
HIV #1	24	M	5.51	716	24	ART-naive	Cervical LN	
HIV #2	30	F	1.6	438	37	ART-naive	Cervical LN	
HIV #3	34	M	5.33	425	17	ART-naive	Cervical LN	
HIV #4	18	M	1.6	504	32	ART-naive	Cervical LN	
HIV #5	31	M	6.3	424	20	ART-naive	Cervical LN	
HIV #6	29	M	4.46	1288	37	ART-naive	Cervical LN	
HIV #7	23	M	5.06	319	25	ART-naive	Cervical LN	
HIV #8	23	M	6.08	315	9	ART-naive	Cervical LN	
HIV #9	28	M	4.65	382	21	ART-naive	Cervical LN	
HIV #10	40	M	5.61	153	11	ART-naive	Cervical LN	
HIV #11	20	F	3.85	504	27	ART-naive	Cervical LN	
HIV #12	22	M	7	381	27	ART-naive	Cervical LN	
HIV #13	19	M	5.13	212	17	ART-naive	Cervical LN	
HIV #14	28	M	4.59	394	24	ART-naive	Cervical LN	
HIV #15	24	M	5.79	290	13	ART-naive	Inguinal LN	
HIV #16	31	M	4.79	406	25	ART-naive	Inguinal LN	
**Neutralizers**								
HIV #17	24	M	5.25	4	1	ART-naive	Cervical LN	
HIV #18	20	M	5.76	175	8	ART-naive	Cervical LN	
HIV #19	40	F	5.05	279	18	ART-naive	Cervical LN	
HIV #20	61	M	5.65	321	7	ART-naive	Cervical LN	
HIV #21	48	M	5.13	415	10	ART-naive	Cervical LN	
HIV #22	31	M	4.58	591	15	ART-naive	Inguinal LN	
HIV #23	38	M	4.85	387	20	ART-naive	Cervical LN	
HIV #24	31	M	4.24	274	22	ART-naive	Cervical LN	
HIV #25	39	M	4.96	179	9	ART-naive	Cervical LN	
HIV #26	38	M	4.76	40	1	ART-naive	Cervical LN	
HIV #27	40	M	5.5	64	7	ART-naive	Cervical LN	
HIV #28	22	M	4.42	135	12	ART-naive	Cervical LN	
HIV #29	23	M	5.13	285	21	ART-naive	Cervical LN	
HIV #30	27	M	4.15	671	21	ART-naive	Inguinal LN	
HIV #31	32	M	3.65	548	18	ART-naive	Inguinal LN	
HIV #32	31	M	4.64	228	12	ART-naive	Inguinal LN	
HIV #33	29	M	5.27	348	12	ART-naive	Cervical LN	
**HIV negative**								
#34_1E	77	M	n/a			n/a	Axillary LN	9mm hyperplasie folliculaire + histiocytose sinusale+
#35_1F	44	M	n/a			n/a	Cervical LN	10mm hyperplasie folliculaire 0/+
#36_1G	52	M	n/a			n/a	Inguinal LN	10mm hyperplasie folliculaire + paracorticale ++
#37_1H	28	F	n/a			n/a	Inguinal LN	11mm hyperplasie folliculaire +
#38_1U	78	M	n/a			n/a	Inguinal LN	8mm hyperplasie folliculaire + Dermatopathic changes+ Congestion+
#39_1J	22	F	n/a			n/a	Cervical LN	

Supplemental Table 2: Study Assays

ID	Tissue Type	Plasma Ab Fingerprinting	Flow	scRNA	mIF_Tfh/B	mIF_Treg	mIF_RNA	HIVseq
**Non neutralizers**								
HIV #1	Cervical LN				●	●	●	●
HIV #2	Cervical LN		●					●
HIV #3	Cervical LN				●	●	●	
HIV #4	Cervical LN							
HIV #5	Cervical LN		●	●				
HIV #6	Cervical LN		●		●	●	●	●
HIV #7	Cervical LN		●					
HIV #8	Cervical LN		●					
HIV #9	Cervical LN				●	●	●	●
HIV #10	Cervical LN		●					●
HIV #11	Cervical LN		●					●
HIV #12	Cervical LN		●		●	●	●	
HIV #13	Cervical LN		●	●	●	●	●	●
HIV #14	Cervical LN		●					●
HIV #15	Inguinal LN			●				●
HIV #16	Inguinal LN			●				●
**Neutralizers**								
HIV #17	Cervical LN	●	●					●
HIV #18	Cervical LN	●	●	●				●
HIV #19	Cervical LN	●	●	●				●
HIV #20	Cervical LN	●	●		●	●	●	●
HIV #21	Cervical LN	●	●		●	●	●	●
HIV #22	Inguinal LN	●			●	●	●	
HIV #23	Cervical LN	●			●		●	
HIV #24	Cervical LN	●			●	●	●	
HIV #25	Cervical LN	●						
HIV #26	Cervical LN	●						
HIV #27	Cervical LN	●						
HIV #28	Cervical LN	●						
HIV #29	Cervical LN	●						
HIV #30	Inguinal LN	●	●	●	●	●	●	●
HIV #31	Inguinal LN	●						
HIV #32	Inguinal LN	●	●	●				
HIV #33	Cervical LN	●						●
**HIV negative**								
#34_1E	Axillary LN				●			
#35_1F	Cervical LN				●			
#36_1G	Inguinal LN				●			
#37_1H	Inguinal LN				●			
#38_1U	Inguinal LN				●			
#39_1J	Cervical LN				●			

Supplemental Table 3: Flow Cytometry Antibodies

Target	Fluorophore	Clone	Manufacturer
**Tfh Panel**			
CD4	BUV805	SK3	BD Biosciences
CD3	H7APC	SK7	BD Biosciences
CD27	A700	M-T271	BD Biosciences
CD45RO	BV786	UCHL1	BD Biosciences
TIM-3 (CD366)	BUV661	7D3	BD Biosciences
CD8	BV570	RPA-T8	Biolegend
CD19	Cy55PE	SJ25C1	Invitrogen
PD-1 (CD279)	BUV496	EH12.1	BD Biosciences
CXCR5 (CD185)	Cy7PE	MU5UBEE	Invitrogen
TIGIT	BB660	MB5A43	BD Biosciences
CD95	BV480	DX2	BD Biosciences
CXCR3 (CD183)	BV650	G025H7	Biolegend
ICOS (CD278)	BV421	DX29	BD Biosciences
PDL-1 (CD274)	BB515	MIH1	BD Biosciences
CD226	BB700	DX11	BD Biosciences
CD57	BB790	NK-1	BD Biosciences
OX40 (CD134)	BV711	L106	BD Horizon
CTLA-4 (CD152)	PE	L3D10	Biolegend
OX40L (CD252)	BUV563	iK-1	BD Biosciences
**Transcription Factor Panel**			
CD4	BV650	SK3	BD Horizon
CD3	H7APC	SK7	BD Biosciences
CD45RO	BV786	UCHL1	BD Biosciences
CD27	BV605	O323	BD Biosciences
PD-1 (CD279)	BV711	EH12.2H7	Biolegend
CD57	BB790	NK-1	BD Biosciences
CD19	ECD	J3-119	Beckman-Coulter
AHR	AF488	polyclonal	R&D systems
CAV-1	PE	Polyclonal	Abcam
TCF1	AF647	7F11A10	Biolegend
CD130	BV421	AM64	BD Biosciences

Supplemental Table 4: Multiparameter Imaging Antibodies

Target	Fluorophore	Species	Isotype	Clone	Manufacturer # Catalog No
**Tfh Panel**					
Bcl-6	Unconjugated	Mouse	IgG1	PG-B6p	DAKO, #M7211
PD-1	Alexa Fluor 488	Mouse	IgG	polyclonal	R&D systems, #FAB7115G
Ki67	Brilliant Violet 421	Mouse	IgG1	B56	BD Horizon, #562899
CD20	eFluor 615	Mouse	IgG2a	L26	eBioscience, #42-0202-82
FoxP3	Alexa Fluor 647	Mouse	IgG1	206D	Biolegend, #320114
CD4	Alexa Fluor 700	Goat	IgG	polyclonal	R&D systems, # FAB8165N
**RNAscope panel**					
PD-1	Unconjugated	Rabbit	IgG	EPR4877	Abcam, #ab137132
FDC	Unconjugated	Mouse	IgM	F3803	SIGMA, #F3803
CD3	Unconjugated	Mouse	IgG1	F7.2.38	DAKO, #M7254
CD20	eFluor 615	Mouse	IgG2a	L26	eBioscience, #42-0202-82
CD4	Alexa Fluor 700	Goat	IgG	polyclonal	R&D systems, # FAB8165N
**Transcription Factor Panel**					
CD25	unconjugated	Mouse	IgG2b	4C9	BioSB, # BSB 6320
IL-10	Alexa Fluor 546	Mouse	IgG2b	E-10	Santa Cruz, #sc-8438
Ki67	Brilliant Violet 480	Mouse	IgG1	B56	BD Horizon, # 566172
CD20	eFluor 615	Mouse	IgG2a	L26	eBioscience, #42-0202-82
FoxP3	Alexa Fluor 647	Mouse	IgG1	206D	Biolegend, #320114
CD4	Alexa Fluor 700	Goat	IgG	polyclonal	R&D systems, # FAB8165N
**Secondary antibodies (all panels)**					
Goat	Alexa Fluor 546	Mouse	IgG1	n/a	Life Technologies, # A21121
Donkey	Brilliant Violet 421	Rabbit	IgG	Poly4046	Biolegend, #406410
Goat	Alexa Fluor 546	Mouse	IgM	n/a	Life Technologies, #A21123
Goat	Alexa Fluor 488	Mouse	IgG2b	n/a	Life Technologies, #A21141
Goat	Alexa Fluor 488	Mouse	IgG1	n/a	Life Technologies, #A21121
